# ULDNA: integrating unsupervised multi-source language models with LSTM-attention network for high-accuracy protein–DNA binding site prediction

**DOI:** 10.1093/bib/bbae040

**Published:** 2024-02-12

**Authors:** Yi-Heng Zhu, Zi Liu, Yan Liu, Zhiwei Ji, Dong-Jun Yu

**Affiliations:** College of Artificial Intelligence, Nanjing Agricultural University, Nanjing 210095, China; School of Computer Science and Engineering, Nanjing University of Science and Technology, Nanjing 210094, China; School of Information Engineering, Yangzhou University, Yangzhou 225000, China; College of Artificial Intelligence, Nanjing Agricultural University, Nanjing 210095, China; School of Computer Science and Engineering, Nanjing University of Science and Technology, Nanjing 210094, China

**Keywords:** protein–DNA interaction, deep learning, unsupervised protein language model, evolution diversity, LSTM-attention network

## Abstract

Efficient and accurate recognition of protein–DNA interactions is vital for understanding the molecular mechanisms of related biological processes and further guiding drug discovery. Although the current experimental protocols are the most precise way to determine protein–DNA binding sites, they tend to be labor-intensive and time-consuming. There is an immediate need to design efficient computational approaches for predicting DNA-binding sites. Here, we proposed ULDNA, a new deep-learning model, to deduce DNA-binding sites from protein sequences. This model leverages an LSTM-attention architecture, embedded with three unsupervised language models that are pre-trained on large-scale sequences from multiple database sources. To prove its effectiveness, ULDNA was tested on 229 protein chains with experimental annotation of DNA-binding sites. Results from computational experiments revealed that ULDNA significantly improves the accuracy of DNA-binding site prediction in comparison with 17 state-of-the-art methods. In-depth data analyses showed that the major strength of ULDNA stems from employing three transformer language models. Specifically, these language models capture complementary feature embeddings with evolution diversity, in which the complex DNA-binding patterns are buried. Meanwhile, the specially crafted LSTM-attention network effectively decodes evolution diversity-based embeddings as DNA-binding results at the residue level. Our findings demonstrated a new pipeline for predicting DNA-binding sites on a large scale with high accuracy from protein sequence alone.

## INTRODUCTION

Protein–DNA interactions are essential for a wide range of biological processes, such as gene expression, DNA replication, chromatin remodeling and signal transduction [1, 2]. Accurate recognition of protein–DNA binding sites is crucial for understanding the molecular mechanisms underlying various biological processes and thus advancing drug discovery [3–6]. Although the current biochemical experiments, such as X-ray crystallography [7] and Cryo-EM [8], are the most precise way for determining DNA-binding sites, they tend to be labor-intensive and time-consuming. Consequently, a large number of sequenced proteins still lack DNA-binding annotations up to now. As of June 2023, the UniProt database [9] contained about 246 million protein sequences, yet less than 0.1% of these sequences had available experimental annotations of DNA-binding sites. There is an immediate need to design efficient computational approaches for predicting protein–DNA binding sites with high accuracy [10–12].

Current methods for DNA-binding site prediction can be categorized into two groups, which are driven by template detection and machine learning, respectively [13]. In the early stage, template detection-based methods were the predominant force in protein–DNA interaction prediction [14, 15]. Specifically, these methods locate DNA-binding sites by detecting the templates with similar sequences or structures to the query. For example, S-SITE [16] identifies sequence templates using PSI-BLAST alignment [17], while PreDNA [18] and DBD-Hunter [19] search templates through designing structure alignment algorithms. Other notable predictors in this field include PreDs [20], DBD-Threader [21], DR_bind [22] and Morozov’s method [23].

A common shortcoming of template detection-based methods is that their accuracy highly depends on the availability of templates with experimentally annotated DNA-binding sites. To overcome this dependency, many machine learning-based methods have been developed. These methods involve extracting manually crafted features from protein sequences and structures (e.g. position-specific scoring matrix [24] and peptide backbone torsion angles [10]), which are further fed to machine learning models (e.g. support vector machine [25] and random forest [26]) to carry out DNA-binding site prediction, including classical examples such as DNAPred [13], TargetDNA [27], MetaDBSite [28] and TargetS [29].

Although machine learning-based methods achieved some progress, their prediction performance was still unsatisfactory. The main reason for this is the shortage of comprehensive and informative feature representations. Specifically, most of these methods are driven by simple and straightforward feature representation methods, such as sequence composition coding and evolution conservation analysis, which fail to capture the complex patterns of protein–DNA interaction [30, 31]. To partially address this challenge, deep learning techniques have been employed in recently proposed DNA-binding site prediction methods, such as Guan’s method [32], PredDBR [33], iProDNA-CapsNet [34] and GraphBind [35]. The significant advantage of deep learning techniques over traditional machine learning methods is that they tend to derive more discriminative feature representations using complicated networks. However, the training efficiency of deep neural network models is frequently constrained by the limited experimental annotation data consisting of only thousands of protein–DNA complexes from the Protein Data Bank (PDB) [36]. As a result, most deep learning models cannot achieve optimal prediction performance.

To relieve the problem arising from the inadequacy of experimentally annotated data, a viable solution is to employ unsupervised protein language models, which are pre-trained on a huge amount of amino acid sequences without DNA-binding annotations via deep learning techniques. Owing to thorough training and learning from extensive sequences, language models could capture crucial inter-residue correlations associated with DNA-binding patterns and encode them as discriminative feature embeddings. Meanwhile, several pre-trained language models have emerged in recent literature, such as TAPE [37] and SeqVec [38]. These methods are frequently employed through supervised deep neural networks in various bioinformatics tasks, including protein design [39, 40], function annotation [41, 42], structure prediction [43, 44] and ligand-binding prediction [45, 46].

In this study, we develop a novelty deep learning model, ULDNA, to accurately predict protein–DNA binding sites through integrating unsupervised protein language models from multiple database sources with the designed LSTM-attention network. Specifically, we utilize three recently proposed language models (i.e. ESM2 [44], ProtTrans [47] and ESM-MSA [48]), separately pre-trained on different large-scale sequence databases, to extract the complementary feature embeddings with evolution diversity, in which the complicated DNA-binding patterns are hidden. Then, an LSTM-attention architecture is specially crafted to effectively decode the evolution diversity-based feature embeddings as the confidence scores of DNA-binding sites at the residue level. ULDNA has been systematically tested on five protein–DNA binding site datasets. Results from computational experiments demonstrated that ULDNA significantly enhances the accuracy of DNA-binding site prediction compared to existing state-of-the-art approaches. The ULDNA online server is freely accessible for academic use through the URL http://csbio.njust.edu.cn/bioinf/uldna/.

## MATERIALS AND METHODS

### Benchmark datasets

The proposed methods were evaluated by five protein–DNA binding site datasets, i.e. PDNA-543, PDNA-41, PDNA-335, PDNA-52 and PDNA-316. PDNA-543 and PDNA-41 were collected by Hu *et al*. [27]. The former is comprised of 543 protein chains with DNA-binding annotations deposited in the PDB database before 10 October 2014, while the latter includes 41 DNA-binding protein chains that were deposited in the PDB after 10 October 2014. Here, the CD-HIT software [49] has been used to eliminate redundant proteins both within and across datasets under a sequence identity cut-off of 30%. PDNA-335 and PDNA-52 were collected by Yu *et al*. [29]. These two datasets consist of 335 and 52 DNA-binding protein chains, respectively, which were released in the PDB before and after 10 March 2010. The sequence identity within each dataset and between different datasets is reduced to 40% through the PISCES software [50]. PDNA-316 was collected by Si *et al*. [28] and composed of 316 DNA-binding chains deposited in the PDB before 31 December 2011, where the sequence identity of any two chains was reduced to 30% using the CD-HIT [49].

Table 1 presents a detailed summary of five datasets, where the definition of DNA-binding sites is described in Text S1 of Supporting Information (SI). Meanwhile, Figure S1 illustrates the frequencies of 20 native amino acids at DNA-binding and non-DNA-binding sites in each dataset.

**Table 1:** Statistical summary of five protein-DNA binding site datasets

Dataset	(Max_L, Min_L, Avg_L)^a^	(Num_DBS, Num_NDBS)^b^
PDNA-543	(1937, 18, 266)	(9549, 134,995)
PDNA-41	(1517, 20, 360)	(734, 14,021)
PDNA-335	(1609, 51, 232)	(6461, 71,320)
PDNA-52	(1132, 54, 331)	(973, 16,225)
PDNA-316	(994, 36, 230)	(5609, 67,109)

^a^Max_L/Min_L/Avg_L: the maximal/minimal/average sequence length.

^b^Num_DBS/Num_NDBS: the number of DNA-binding sites/non-DNA-binding sites.

### The architecture of ULDNA

**Figure 1:**
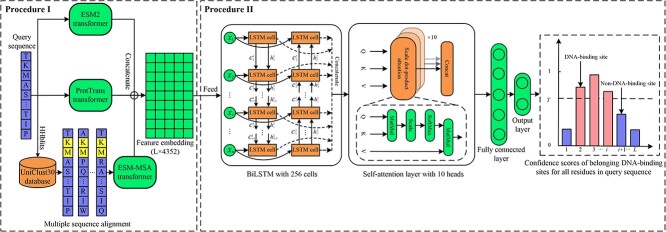
The workflow of ULDNA.

As depicted in Figure 1, ULDNA is a deep learning model for predicting protein–DNA binding sites, where the input is a query sequence with amino acids and the output includes the confidence scores of DNA-binding sites at the residue level. ULDNA comprises two procedures, i.e. (i) feature embedding extraction using multi-source language models and (ii) DNA-binding site prediction using the LSTM-attention network.

#### Procedure I: feature embedding extraction using multi-source language models

The input sequence is fed to ESM2 [44] and ProtTrans [47] transformers to generate two feature embedding matrices with the scales of $L\times 2560$ and $L\times 1024$, respectively. Meanwhile, we search the multiple sequence alignment (MSA) of the input sequence from the UniClust30 database [51]. This MSA is subsequently fed to the ESM-MSA transformer [48] to generate another feature embedding matrix with the scale of $L\times 768$. Here, $L$ is the length of the input sequence, 2560, 1024 and 768 are preset hyper-parameters in transformer models. ESM2, ProtTrans, and ESM-MSA are both unsupervised attention networks with 36, 24 and 12 layers, respectively, and trained on Uniref50 [52], UniClust30 & Uniref50, and BFD (Big Fantastic Database) [53] & Uniref50 databases, respectively, where ‘&’ means that two databases are both used to train a transformer. Each transformer has learned abundant evolution knowledge from millions of sequences and could encode the input sequence (or MSA) as a feature embedding matrix with evolution diversity. Considering that the evolution knowledge from multiple database sources could be complementary, we concatenate the above-mentioned three feature embedding matrices from different transformer models as a hybrid embedding matrix with the scale of $L\times 4352$.

#### Procedure II: DNA-binding site prediction using the LSTM-attention network

The hybrid feature embedding is fed to the designed LSTM-attention network to generate a score vector with $L$ dimensions, indicating the confidence scores of belonging to DNA-binding sites for all residues in the query sequence. In the LSTM-attention network, a BiLSTM layer and a self-attention layer are combined to further strengthen the relationship between evolution diversity-based feature embeddings and DNA-binding patterns at the residue level to improve prediction accuracy.

### Unsupervised protein language models

ESM2 transformer [44] is an unsupervised deep attention neural network with 36 layers, as depicted in Figure S2. Here, the input of ESM2 is a query sequence with amino acids, while the output is an evolution diversity-based feature embedding matrix. Each layer consists of 20 attention heads and a feed-forward network (FFN), where each head performs the scale dot-product operation to learn the evolution correlation between amino acids in the query sequence from an individual view. Meanwhile, the FFN fuses the evolution knowledge from all attention heads to capture the evolution diversity for the entire sequence. The ESM2 model with 3 billion parameters was trained on over 60 million proteins from the UniRef50 database, as carefully described in Text S2 of SI.

ProtTrans transformer [47] shares a similar architecture to the ESM2. This model is composed of 24 attention layers, with each layer including 32 attention heads. The ProtTrans model with 3 billion parameters was trained on over 45 million proteins from BFD and UniRef50 databases.

ESM-MSA transformer [48] aims to capture the co-evolution knowledge for the input MSA and encode it as a feature embedding matrix, as shown in Figure S3. ESM-MSA consists of 12 attention blocks, with each block including one row-attention layer and one column-attention layer that separately learn the co-evolution correlation between amino acids at the sequence and position level. The ESM-MSA model with 100 million parameters was trained on over 26 million MSAs from Unclust30 and UniRef50 databases, with details in Text S3.

### LSTM-attention network

The designed LSTM-attention network includes a BiLSTM layer, a self-attention layer, a fully connected layer, and an output layer, as shown in Figure 1. The BiLSTM includes a forward LSTM and a backward LSTM, which have the same architecture consisting of 256 cells with reverse propagation directions. Each LSTM cell is mainly composed of two states (i.e. cell state $c$ and hidden state $h$) and three gates (i.e. forget gate $f$, input gate $i$ and output gate $o$). The cell and hidden states are separately used to store and output the signals at the current time-step. The forget, input and output gates are used to control the ratios of incorporating the history signal, inputting the current signal and outputting the updated signal, respectively. Specifically, at time-step $t$ ($t\le L$, $L$ is the length of the input sequence), the above-mentioned states and gates are computed as follows:


(1) \begin{equation*} {h}_t={o}_t\cdot \tanh \left({c}_t\right) \end{equation*}

(2) \begin{equation*} {c}_t={f}_t\cdot{c}_{t-1}+{i}_t\cdot{c}_t^{\prime } \end{equation*}

(3) \begin{equation*} {c}_t^{\prime }=\tanh \left({w}_c\cdot \left[{h}_{t-1},{x}_t\right]+{b}_c\right) \end{equation*}

(4) \begin{equation*} {o}_t=\sigma \left({w}_o\cdot \left[{h}_{t-1},{x}_t\right]+{b}_o\right) \end{equation*}

(5) \begin{equation*} {f}_t=\sigma \left({w}_f\cdot \left[{h}_{t-1},{x}_t\right]+{b}_f\right) \end{equation*}

(6) \begin{equation*} {i}_t=\sigma \left({w}_i\cdot \left[{h}_{t-1},{x}_t\right]+{b}_i\right) \end{equation*}

where ${c}_{t-1}$ and ${h}_{t-1}$ are cell state and hidden state, respectively, at the time-step $t-1$, ${x}_t$ is the input at the time-step $t$ (i.e. the feature embedding vector with 4352 dimensions of the $t$th residue in the query sequence for DNA-binding prediction), ${w}_{\ast }$ is the weight, ${b}_{\ast }$ is the bias, $\left[,\right]$ is concatenation operation between two vectors and $\sigma \left(\cdot \right)$ is the Sigmoid function. The output of the BiLSTM layer is represented as a $L\times 512$ matrix through concatenating the hidden states in all LSTM cells at all time-steps.

The self-attention layer consists of 10 attention heads, each of which performs the scale dot-product attention as follows:


(7) \begin{equation*} {A}_i= SoftMax\left({M}_i^Q\cdot{\left({M}_i^K\right)}^T/\sqrt{d_i}\right)\cdot{M}_i^V \end{equation*}

(8) \begin{equation*} {M}_i^Q=H\cdot{W}_i^Q,{M}_i^K=H\cdot{W}_i^K,{M}_i^V=H\cdot{W}_i^V \end{equation*}

where $H$ is the output matrix by the BiLSTM; ${A}_i$ is an attention matrix in the $i$th attention head; ${M}_i^Q$, ${M}_i^K$ and ${M}_i^V$ are Query, Key and Value matrices with the scale of $512\times 64$, respectively; ${M}_i^Q\cdot{M}_i^K$ is an $L\times L$weight matrix measuring the position-correlation of amino acid pairs in the query; and ${d}_i$ is a scale factor.

The attention matrices in all 10 heads are concatenated and then inputted into the fully connected layer containing 1024 neurons, followed by an output layer with one neuron:


(9) \begin{equation*} A={A}_1{A}_2\dots{A}_{10} \end{equation*}

(10) \begin{equation*} F= Relu\left({W}_a\cdot A+{b}_a\right) \end{equation*}

(11) \begin{equation*} s=\sigma \left({W}_s\cdot F+{b}_s\right) \end{equation*}

where $Relu\left(\cdot \right)$ is the linear rectification function and $s$ is a score vector with $L$ dimensions, indicating the confidence scores of belonging to DNA-binding sites for all residues in the query sequence.

### Loss function

We use the cross-entropy loss [54] as the training loss of ULDNA:


(12) \begin{equation*} Loss=\frac{1}{L}\cdot \sum_{i=1}^L\left({y}_i\cdot \log \left({s}_i\right)+\left(1-{y}_i\right)\cdot \log \left(1-{s}_i\right)\right) \end{equation*}

where ${s}_i$ is the confidence score of belonging to the DNA-binding site at the $i$th residue in the query sequence; ${y}_i=1$, if the $i$th residue is a DNA-binding site annotated by experimental protocols; otherwise, ${y}_i=0$. The training loss is minimized to optimize the hyper-parameters of the ULDNA model via the Adam optimization algorithm [55], where the learning rate, dropout rate and batch size are set to be 0.001, 0.2 and 1, respectively.

### Implementation details

The five benchmark datasets were constructed by three individual works [27–29], leading to different definitions of protein–DNA binding sites (see details in Text S1 of SI). Therefore, we should use the datasets with the same definition of DNA-binding sites for training and testing the proposed ULDNA model. Specifically, we separately used PDNA-543 and PDNA-335 datasets to train models, which were then tested on PDNA-41 and PDNA-52, respectively, under independent validation. Moreover, there is no overlap between the two test datasets, because the release dates of proteins in the PDNA-52 are both earlier than those of proteins in the PDNA-41 (see details in the section of ‘Benchmark datasets’). The hyper-parameters and thresholds of the ULDNA model were determined on the corresponding training dataset under 10-fold cross-validation. In addition, the PDNA-316 dataset was used to further evaluate the performance of ULDNA over 10-fold cross-validation.

In the 10-fold cross-validation, the dataset was randomly split into 10-folds at the sequence level. Then, 9-folds were used to train the model, which was tested on the remaining 1-fold. This process was repeated 10 times, ensuring that each protein residue in the dataset was assigned a confidence score belonging to the DNA-binding site. Finally, an appropriate threshold was selected to evaluate the overall prediction performance of the model on the entire dataset under 10-fold cross-validation, where a protein residue was predicted as the DNA-binding site if its confidence score was higher than the selected threshold. To maximize the prediction performance of models over cross-validation, we optimized the hyper-parameters of models, e.g. the number of attention heads and training epochs, using the grid search strategy.

To reduce the influence of randomness, we repeatedly train the model 10 times and then use the average of the confidence scores outputted by 10 models as the final score for each protein residue.

### Evaluation indices

Four indices are used to evaluate the performance of the proposed methods, including Sensitivity (Sen), Specificity (Spe), Accuracy (Acc) and Mathew’s Correlation Coefficient (MCC):


(13) \begin{equation*} Sen= TP/\left( TP+ FN\right) \end{equation*}

(14) \begin{equation*} Spe= TN/\left( TN+ FP\right) \end{equation*}

(15) \begin{equation*} Acc=\left( TP+ TN\right)/\left( TP+ FP+ TN+ FN\right) \end{equation*}

(16) \begin{equation*} MCC\!=\!\left( TP\!\times\! TN\!-\! FP\!\times\! FN\right)/\sqrt{\left( TP+ FP\right)\left( TN+ FN\right)\left( TP+ FN\right)\left( TN+ FP\right)} \end{equation*}

where TP, TN, FP and FN separately stand for numbers of true positives, true negatives, false positives and false negatives.

Since the four indices mentioned above depend on the preset threshold, choosing a suitable threshold is crucial for making fair comparisons between different models. In this study, the reported evaluation indices of the ULDNA model are determined by the threshold that yields the maximum MCC value on the training dataset under 10-fold cross-validation, unless stated otherwise. In addition, to evaluate the overall prediction performance of models, a threshold-independent index is utilized, i.e. the area under the receiver operating characteristic curve (AUROC) [56].

## RESULTS AND DISCUSSION

### Comparison with existing protein–DNA binding site predictors

To demonstrate the strong performance of the proposed ULDNA, we made a comparison with 12 existing popular DNA-binding site predictors, including BindN [57], ProteDNA [58], BindN+ [59], MetaDBSite [28], DP-Bind [60], DNABind [61], TargetDNA [27], iProDNA-CapsNet [34], DNAPred [13], Guan’s method [32], COACH [16] and PredDBR [33], on the PDNA-41 test dataset under independent validation, as summarized in Table 2.

**Table 2:** Performance comparisons between ULDNA and 12 competing predictors on the PDNA-41 test dataset under independent validation

Method	Sen	Spe	Acc	MCC	AUROC
BindN^a^	0.456	0.809	0.792	0.143	-
ProteDNA^a^	0.048	0.998	0.951	0.160	-
BindN+ ($Spe\approx 0.95$)^a^	0.241	0.951	0.916	0.178	-
BindN+ ($Spe\approx 0.85$)^a^	0.508	0.854	0.837	0.213	-
MetaDBSite^a^	0.342	0.934	0.904	0.221	-
DP-Bind^a^	0.617	0.824	0.814	0.241	-
DNABind^a^	0.702	0.803	0.798	0.264	-
TargetDNA ($Sen\approx Spe$)^a^	0.602	0.858	0.845	0.269	-
TargetDNA ($Spe\approx 0.95$)^a^	0.455	0.933	0.909	0.300	-
iProDNA-CapsNet ($Sen\approx Spe$)^b^	0.753	0.753	0.753	0.245	-
iProDNA-CapsNet ($Spe\approx 0.95$)^b^	0.422	0.949	0.924	0.315	-
DNAPred ($Sen\approx Spe$)^c^	0.761	0.767	0.761	0.260	0.858
DNAPred ($Spe\approx 0.95$)^c^	0.447	0.949	0.924	0.337	0.858
Guan’s method^d^	0.476	0.964	0.949	0.357	-
COACH^e^	0.462	0.951	0.927	0.352	-
PredDBR ($Sen\approx Spe$)^e^	0.764	0.758	0.758	0.264	-
PredDBR ($Spe\approx 0.95$)^e^	0.431	0.958	0.931	0.351	-
PredDBR (threshold = 0.5)^e^	0.391	0.968	0.939	0.359	-
ULDNA ($Sen\approx Spe$)	0.824	0.899	0.895	0.458	0.935
ULDNA ($Spe\approx 0.95$)	0.556	0.970	0.950	0.499	0.935
ULDNA (threshold = 0.5)	0.271	0.994	0.958	0.417	0.935

^a, b, c, d, e^Results excerpted from TargetDNA [29], iProDNA-CapsNet [36], DNAPred [14], Guan et al [34] and PredDBR [35], respectively; ‘$Sen\approx Spe$’ and ‘$Spe\approx 0.95$’ mean that the thresholds make $Sen\approx Spe$ and $Spe\approx 0.95$, respectively, on the PDNA-543 training dataset over 10-fold cross-validation. ‘-’ means that the corresponding value is unavailable.

It is observed that ULDNA obtains the highest MCC values among all 13 competing methods. Compared to the second best performer PredDBR (a recently proposed deep learning model), ULDNA gains 13.3% improvement of MCC values on average under three different thresholds. More importantly, four evaluation indices of ULDNA are both higher than those of PredDBR under $Sen\approx Spe$ and $Spe\approx 0.95$. Meanwhile, a similar trend but with more significant distinctions can be observed in comparison with other predictors. Taking DNAPred as an example, ULDNA shares the improvements of 6.3, 13.2, 13.4, 19.8 and 7.7%, respectively, on Sen, Spe, Acc, MCC and AUROC values under $Sen\approx Spe$. It cannot escape from our notice that ProteDNA gains the highest Spe (0.998) but with the lowest Sen (0.048). This is due to that ProteDNA predicts too many false negatives.

Table 3 illustrates the performance comparison among ULDNA, DNABR [31], MetaDBSite [28], TargetS [29], DNAPred [13], COACH [16] and PredDBR [33] on the PDNA-52 test dataset under independent validation, where ULDNA achieves the highest MCC value among all control methods. Specifically, the improvements in MCC values between ULDNA and the other 6 predictors range from 6.6 to 33.2%.

**Table 3:** Performance comparisons between ULDNA and 6 competing predictors on the PDNA-52 test dataset under independent validation

Method	Sen	Spe	Acc	MCC	AUROC
DNABR^a^	0.407	0.873	0.846	0.185	-
MetaDBSite^a^	0.580	0.764	0.752	0.192	-
TargetS^a^	0.413	0.965	0.933	0.377	0.836
DNAPred^b^	0.518	0.949	0.925	0.405	0.876
COACH^c^	0.599	0.935	0.916	0.420	-
PredDBR^c^	0.539	0.958	0.935	0.451	-
ULDNA	0.704	0.944	0.931	0.517	0.945

^a, b, c^Results excerpted from TargetS [31], DNAPred [14] and PredDBR [35]. ‘-’ means that the corresponding value is unavailable.

We further compare our method with all the above-mentioned methods as well as the other 4 competing methods, including EC-RUS [62], DBS-PRED [63], DISIS [64] and BindN-rf [30], on three training datasets (i.e. PDNA-543, PDNA-335 and PDNA-316) under 10-fold cross-validation, as listed in Tables S1, S2 and S3 of SI. Again, the proposed ULDNA outperforms all other methods.

### Contribution analysis of different protein language models

**Figure 2:**
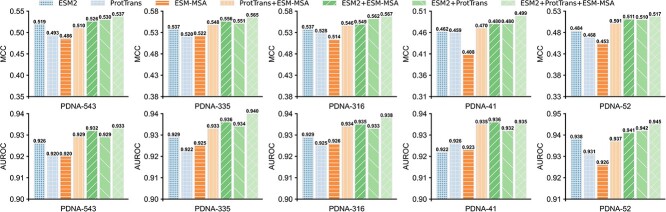
The MCC and AUROC values of seven feature embeddings on five benchmark datasets.

The contributions of three employed protein language models, i.e. ESM2, ProtTrans and ESM-MSA, could be analyzed by further benchmarking the performance of the designed LSTM-attention network with seven different feature embeddings, respectively. These include three individual embeddings extracted from ESM2, ProtTrans and ESM-MSA, and four hybrid embeddings generated by ProtTrans + ESM-MSA (PE), ESM2 + ESM-MSA (EE), ESM2 + ProtTrans (EP) and ESM2 + ProtTrans + ESM-MSA (EPE = ULDNA). Here, ‘+’ indicates that we directly concatenate individual embeddings of different language models as a hybrid embedding. Figure 2 presents the performance comparison between seven feature embeddings across three training datasets (PDNA-543, PDNA-335 and PDNA-316) under 10-fold cross-validation and two test datasets (PDNA-41 and PDNA-52) under independent validation, where the *P*-values of MCC and AUROC values between EPE and other six feature embeddings under two-sided Student’s *t*-test [65] are listed in Tables S4 and S5 and discussed in Text S4 of SI.

It could be found that EPE achieves the best performance among the seven feature embeddings. From the view of MCC values, EPE gains the average improvements of 2.9, 4.3, 6.0, 2.2, 1.3 and 1.0% on five datasets in comparison with ESM2, ProtTrans, ESM-MSA, PE, EE and EP, respectively, where the *P*-values are both below 0.05 for all the comparisons. With respect to AUROC values, EPE occupies the top-1 position on four out of five datasets. Moreover, ESM2 shows the highest MCC and AUROC values among three individual embeddings; Meanwhile, the largest increase is caused by adding ESM2 to PE on each dataset.

These data demonstrate the following two conclusions. First, three language models pre-trained on different sequence database sources are complementary for improving DNA-binding site prediction. Second, ESM2 makes the most important contribution among the three language models.

### Ablation study

We designed an ablation study to investigate the impact of algorithmic advancements in the ULDNA on its enhanced performance. Specifically, we began with a baseline model M0 and progressively incorporated ULDNA’s algorithmic elements to implement two improved models M1 and M2, where M2 is equivalent to ULDNA. The architectures of the three ablation models are depicted in Figure S4, with the following procedures.

#### M0

This model is built on the BiLSTM architecture, which is serially composed of a BiLSTM layer with 256 cells, a fully connected layer with 1024 neurons, and an output layer with one neuron. Meanwhile, the activation functions in the last two layers are employed by the linear rectification function and Sigmoid function, respectively. Here, the input sequence is encoded as the one-hot coding matrix [66], which is then fed to the BiLSTM architecture to output the confidence scores of belonging to DNA-binding sites for all residues. In addition, the loss function is designed as the cross-entropy loss, as shown in Equation (12).

#### M1

The one-hot coding matrix used in M0 is replaced by the hybrid feature embedding matrix concatenated by three individual embeddings from the ESM2, ProtTrans and ESM-MSA transformers. This hybrid embedding is further fed to the BiLSTM architecture employed by M0 to output the confidence scores of DNA-binding sites.

#### M2 (M2 = ULDNA)

We add a self-attention layer consisting of 10 attention heads after the BiLSTM layer in M1.

**Figure 3:**
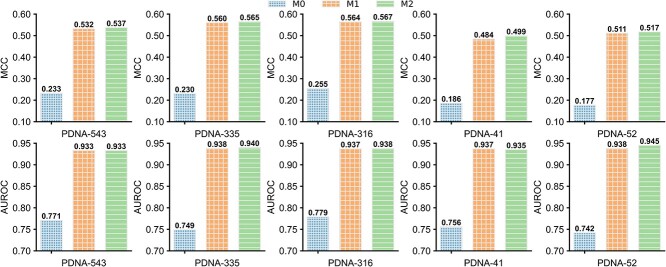
The MCC and AUROC values of three ablation models on five benchmark datasets.

Figure 3 summarizes the performances of three ablation models across three training datasets under 10-fold cross-validation and two test datasets under independent validation. In comparison with M0, M1 shows a great performance improvement, with the MCC and AUROC values averagely rising by 31.4 and 17.7%, respectively, on five benchmark datasets. This observation demonstrates the significant importance of protein language models for improving DNA-binding site prediction. The performance advantage of M1 over M0 is mainly attributed to that the employed transformers learn the abundant knowledge, highly associated with protein–DNA interaction patterns, from complementary sequence database sources. After adding the self-attention layer, M2 achieves an average increase of 0.7% in MCC values on five datasets in contrast to M1. Although the AUROC values of M2 are slightly lower than those of M1 across the PDNA-543 and PDNA-41, they consistently increase on the other three datasets. These findings suggest that the inclusion of the self-attention layer helps improve the overall accuracy of DNA-binding site prediction, albeit to a lesser extent compared to the enhancements provided by protein language models.

### Testing on recently released PDB targets

The proposed ULDNA was further compared with nine existing DNA-binding site prediction methods on 136 recently released DNA-binding protein chains from the PDB database, including DP-Bind [60], TargetS [29], TargetDNA [27], DNAPred [13], GraphBind [35], NCBRPred [67], GraphSite [10], PredDBR [33] and iDRNA-ITF [68]. Specifically, we collected 1096 DNA-binding protein chains under a cut-off of 30% sequence identity, where the 960 chains and 136 chains separately released in the PDB before and after 1 January 2023 were used as the training dataset (i.e. PDNA-960) and test dataset (i.e. PDNA-136), respectively, for the ULDNA model (see details in Text S5 of SI). Meanwhile, for the nine existing predictors, we downloaded the standalone software (or accessed the computation platforms) and implemented them on the PDNA-136 dataset using the default settings. Moreover, considering the unbalanced distribution of DNA binding sites, we added a new evaluation index, i.e. average precision (AP, see details in Text S6), in all comparisons.

Table 4 summarizes the prediction performance of ULDNA and 9 competing predictors on the PDNA-136 test dataset. It could be found that the proposed ULDNA achieves the best performance among 10 predictors in terms of MCC, AUROC and AP values. Meanwhile, the Sen and Spe values of ULDNA are separately ranked 3 and 2. Compared to the second-best performer GraphSite learning DNA-binding patterns from feature embeddings of AlphaFold2 [69], our method achieves 6.1, 5.8 and 1.6% improvement for MCC, AP and AUROC values, respectively. Moreover, ULDNA is ranked number 1 for all seven evaluation indices in comparison with TargetS, TargetDNA, DNAPred, NCBRPred, GraphSite and PredDBR.

**Table 4:** Performance comparisons between ULDNA and nine state-of-the-art predictors on the PDNA-136 test dataset under independent validation

Method	Sen	Spe	Acc	MCC	AP	AUROC
DP-Bind	0.622	0.787	0.779	0.199	0.144	-
TargetS	0.266	0.959	0.929	0.211	0.264	-
TargetDNA	0.455	0.907	0.886	0.238	0.209	0.802
DNAPred	0.432	0.934	0.912	0.275	0.260	0.820
GraphBind	0.628	0.925	0.911	0.379	0.303	0.898
NCBRPred	0.372	0.947	0.921	0.261	0.203	0.799
GraphSite	0.541	0.950	0.931	0.390	0.302	0.907
PredDBR	0.351	0.947	0.920	0.246	0.234	0.775
iDRNA-ITF	0.325	0.966	0.937	0.282	0.208	-
ULDNA	0.544	0.965	0.947	0.451	0.360	0.923

For each competing predictor, we used the default threshold in the corresponding program to calculate evaluation indices. ‘-’ means that the AUROC value is unavailable due to that the corresponding predictor can only output the binary prediction results (‘0’ and ‘1’) rather than confidence scores.

### Case study

**Figure 4:**
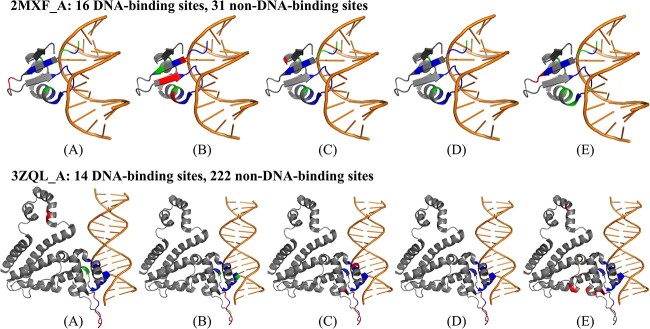
Visualization of prediction results for two proteins (2MXF_A and 3ZQL_A) using five DNA-binding site prediction models: (A) LA-ESM2, (B) LA-ProtTrans, (C) LA-ESM-MSA, (D) ULDNA, (E) PredDBR. The atomic-level native structure of each protein is downloaded from the PDB database and then plotted as the cartoon picture using PyMOL software [70]. The color scheme is used as follows: DNA in orange, true positives in blue, false positives in red and false negatives in green.

To delve deeper into the effects of different DNA-binding site prediction approaches, we chose two proteins with PDB IDs of 2MXF_A and 3ZQL_A from our test datasets as case examples. For each protein, we used four in-house methods (denoted as LA-ESM2, LA-ProtTrans, LA-ESM-MSA and ULDNA) and a competing method (PredDBR [33]) to predict the corresponding DNA-binding sites. Four in-house methods use the same LSTM-attention network with different feature embeddings from ESM2, ProtTrans, ESM-MSA and ESM2 + ProtTrans+ESM-MSA, respectively. Here, ‘+’ indicates that we directly concatenate individual embeddings of different language models as a hybrid embedding. Table 5 summarizes the modeling results of two proteins for five DNA-binding site prediction methods, where the corresponding visualization results are illustrated in Figure 4. In addition, the predicted and native DNA-binding sites of two proteins by five methods are listed in Table S6 of SI.

**Table 5:** The modeling results of five DNA-binding site prediction methods on two representative examples

Method	2MXF_A					3ZQL_A				
	TP	FP	TN	FN	MCC	TP	FP	TN	FN	MCC
LA-ESM2	12	2	29	4	0.710	12	9	213	2	0.678
LA-ProtTrans	12	4	27	4	0.621	11	8	214	3	0.651
LA-ESM-MSA	12	1	30	4	0.760	14	10	212	0	0.746
ULDNA	13	0	31	3	0.861	14	8	214	0	0.783
PredDBR	8	2	29	6	0.564	14	18	204	0	0.634

From the experiment data, we observed several interesting phenomena. First, the protein language models are critical to improve DNA-binding site prediction. Specifically, four in-house methods with pre-trained protein language models both show higher MCC values than the competing PredDBR without language models on two proteins. Taking ULDNA as an example, it gains the MCC increases by 29.7 and 14.9%, respectively, on 2MXF_A and 3ZQL_A in comparison with PredDBR.

Second, the combination of complementary protein language models can further increase the accuracy of ULDNA. In 2MXF_A, three in-house methods (i.e. LA-ESM2, LA-ProtTrans and LA-ESM-MSA) with different language models detect a total of 14 true positives. This number surpasses the true positives predicted by each individual method, suggesting that three language models (i.e. ESM2, ProtTrans and ESM-MSA) extract complementary knowledge from multiple sequence databases. Meanwhile, the false positives predicted by one in-house method can be corrected by the other two methods. For example, LA-ESM2 generates two false positives (10P and 11H), which are correctly predicted as non-DNA-binding sites by LA-ProtTrans and LA-ESM-MSA. As a result, by taking the combination of three language models, ULDNA gains the most true positives without false positives among all methods. Occasionally, one in-house method could capture all the true positives identified by other methods. In the case of 3ZQL_A, LA-ESM-MSA encompasses all the true positives predicted by both LA-ESM2 and LA-ProtTrans. Despite this overlap, the overall accuracy of the final ULDNA is still improved by including all individual methods to reduce false positives.

## CONCLUSIONS

In this work, a novelty deep learning model, ULDNA, is developed to predict DNA-binding sites from protein sequences through leveraging an LSTM-attention architecture embedded with protein language transformer models. The results from benchmark testing have shown that ULDNA significantly surpasses existing popular methods in the accuracy of predicting DNA-binding sites. The performance enhancement of ULDNA stems from two advancements. First, three transformer models pre-trained on multiple large-scare sequence databases could capture the complementary feature embeddings with evolution diversity, which are highly associated with protein–DNA interactions. Second, the specifically designed LSTM-attention network further strengthens the relationship between evolution diversity-based feature embeddings and DNA-binding patterns to improve prediction accuracy.

Although the prediction performance is promising, there remains substantial potential for further advancements. First, the serial feature concatenation strategy, currently utilized in the ULDNA, cannot perfectly deal with the redundant information among the feature embeddings from different transformers. Thus, designing a more advanced approach to feature fusion could help reduce the adverse effects arising from information redundancy in the future. Second, with the development of protein structure prediction models (e.g. AlphaFold2 [69] and ESMFold [44]), the predicted structures will have the huge potential for improving DNA-binding site prediction. Resarches in these directions are currently ongoing.

**Key Points**

- Accurate recognition of protein–DNA binding sites is crucial for understanding the molecular mechanisms underlying various biological processes and thus advancing drug discovery. This study has designed a novelty deep learning model ULDNA to accurately predict DNA-binding sites from protein sequences through integrating three unsupervised protein language models from multiple database sources with the designed LSTM-attention network.
- Results from computational experiments have revealed that ULDNA significantly surpasses existing popular methods in the accuracy of DNA-binding site prediction. The major strength of ULDNA stems from employing three transformer language models that can effectively capture complementary feature embeddings with evolution diversity that are highly associated with complicated DNA-binding patterns.
- An online server for predicting protein–DNA binding sites is freely accessible through the URL http://csbio.njust.edu.cn/bioinf/uldna/.

## Supplementary Material

SI_bbae040

## CODE AVAILABILITY

The datasets and source codes can be freely downloaded at https://github.com/yiheng-zhu/ULDNA.

## Author Biographies

**Yi-Heng Zhu** received his PhD degree in control science and engineering from Nanjing University of Science and Technology in 2023. He is currently a lecturer at the College of Artificial Intelligence, Nanjing Agricultural University. His research interests include bioinformatics, machine learning and pattern recognition.

**Zi Liu** received his PhD degree from the School of Computer Science and Engineering, Nanjing University of Science and Technology. From 2017 to 2019, he participated in a joint PhD program at the prestigious University of Michigan, USA. He is a lecturer at the School of Information and Engineering, Jingdezhen Ceramic University. His research interests include bioinformatics, machine learning and pattern recognition.

**Yan Liu** received his PhD degree in computer science from Nanjing University of Science and Technology in 2023. He is currently a young hundred distinguished professor at the School of Information Engineering, Yangzhou University, China. His research interests include pattern recognition, machine learning and bioinformatics.

**Zhiwei Ji** joined in the School of Artificial Intelligence in Nanjing Agricultural University (NJAU) on 21 August 2020 as a full professor. Prior to this position, he was an assistant professor at the University of Texas Health Science Center at Houston (UTHealth). He has been working on Systems Biology, Bioinformatics, Pattern Recognition, Big Data Analysis and Modeling for over 10 years. Currently, he is the director of Center for Data Science and Intelligent Computing at NJAU.

**Dong-Jun Yu** is a full professor at the School of Computer Science and Engineering, Nanjing University of Science and Technology, Nanjing, China. His research interests include pattern recognition, machine learning and bioinformatics. He is a senior member of the China Computer Federation (CCF) and a senior member of the China Association of Artificial Intelligence (CAAI).
