# Supplementary material for: ULDNA: integrating unsupervised multi-source language models with LSTM-attention network for high-accuracy protein–DNA binding site prediction
Source: Brief Bioinform. 2024 Feb 12;25(2):bbae040. doi: 10.1093/bib/bbae040 (PMC10939370; doi:10.1093/bib/bbae040)
Supplement: SI_bbae040 [file si_bbae040.docx]

**Supplementary Materials**

**Table of content**

**Supporting Text**

- Text S1. Definitions of DNA-binding sites.
- Text S2. Procedures for the ESM2 transformer.
- Text S3. Procedures for the ESM-MSA transformer.
- Text S4. *p*-value calculation between EPE and the other six feature embeddings.
- Text S5. Procedures for constructing PDNA-960 and PDNA-136 datasets.
- Text S6. Formulas for calculating average precision.

**Supporting Figures**

- Figure S1. The frequency distributions of 20 native amino acids among DNA-binding and non-DNA-binding sites for five benchmark datasets.
- Figure S2. The workflow of the ESM2 transformer.
- Figure S3. The workflow of the ESM-MSA transformer.
- Figure S4. The architectures of three ablation models.

**Supporting Tables**

- Table S1. The performance of five DNA-binding site predictors on PDNA-543 over ten-fold cross-validation.
- Table S2. The performance of five DNA-binding site predictors on PDNA-335 over ten-fold cross-validation.
- Table S3. The performance of eleven DNA-binding site predictors on PDNA-316 over ten-fold cross-validation.
- Table S4. The *p*-values of MCC values between EPE and the other six feature embeddings on five benchmark datasets, where the base model is the designed LSTM-attention network.
- Table S5. The *p*-values of AUROC values between EPE and the other six feature embeddings on five benchmark datasets, where the base model is the designed LSTM-attention network.
- Table S6. The predicted and native DNA-binding sites of two representative proteins for five DNA-binding prediction methods.

**Supporting Text**

**Text S1. Definitions of DNA-binding sites**

In the PDNA-335 and PDNA-52 datasets, the DNA-binding sites are defined using the criterion of the protein-ligand binding database BioLip [1], consistent with that used in the Critical Assessment of Structure Prediction (CASP) [2, 3]. Specifically, a protein residue with at least an inter-molecular atomic contact to a DNA molecule is labeled as a DNA binding site, where the inter-molecular atomic contact is a non-hydrogen protein-DNA atom pair whose Euclidian distance is less than the sum of van der Waals radii plus 0.5 Å. It is noted that this criterion has been tightened up in the newest BioLip database (i.e., BioLip2) [4], in which a protein residue with at least two inter-molecular atomic contacts to a DNA is defined as a DNA binding site.

In the PDNA-316 dataset, the DNA-binding sites are defined by the criteria of Ahmad’s work [5], which is the first work for protein-DNA binding site prediction (to our best knowledge). Specifically, in a protein-DNA complex, an amino acid residue in the protein is defined as a DNA-binding site if the distance between any atoms of this residue and any atoms of the DNA molecule is less than a cut-off value. In the PDNA-316, this cut-off is set to be 3.5 Å.

PDNA-543 and PDNA-41 were constructed by the TargetDNA paper [6], which does not provide any details for defining DNA-binding sites. However, we infer that the definition of DNA-binding sites for these two datasets is the same as that for the PDNA-316 dataset via the criteria of Ahmad’s work due to the following observations. Specifically, there are 101 overlap proteins between the PDNA-543 and PDNA-316 datasets, where 98.0% overlap proteins have consistent DNA-binding site annotations between these two datasets. Meanwhile, there are 91 overlap proteins between the PDNA-543 and PDNA-335 datasets, but only 12.1% overlap proteins have consistent DNA-binding site annotations.

To further demonstrate our inference, we designed the following computational experiments. Specifically, we separately used different criteria to re-define the DNA-binding sites for the protein chains in the PDNA-543 and PDNA-41 datasets and then calculated the consistency ratio between re-defined DNA-binding sites and originally annotated DNA-binding sites. The higher consistency ratio means that the re-implemented criterion is more similar to the original criterion used in the TargetDNA paper. It is noted that there are 86 protein chains whose DNA-binding sites cannot be re-defined in the PDNA-543 and PDNA-41. The underlying reason is that these proteins have been obsoleted or updated in the PDB database from the year 2016 to now. As a result, their PDB structures are unavailable, or the sequences extracted from their structures are inconsistent with the original sequences provided by the TargetDNA paper. Therefore, we only collected 498 protein chains from the PDNA-543 and PDNA-41 whose consistency ratio between re-defined and originally annotated DNA-binding sites could be calculated. Here, the consistency ratio (denoted as CR) is defined as CR=N1/N2, where N1 is the number of protein chains whose re-defined DNA-binding sites are consistent with the originally annotated DNA-binding sites in the TargetDNA paper; N2 is the total number of available protein chains (i.e., N2=498). In our experiments, we used four different criteria to re-define DNA-binding sites, including the criterion of CASP and the criterion of Ahmad’s work with the cutoff values of 3.0 Å, 3.5 Å, and 4.0 Å, where the corresponding CR values are 9.8%, 0.0%, 89.6%, and 4.2%, respectively. These experiment data further demonstrated that the TargetDNA paper probably used the criterion of Ahmad’s work with the cut-off=3.5 Å to define DNA-binding sites. It could not escape our notice that there is nearly a 10% difference between the re-defined and originally annotated DNA-binding sites. This difference may be due to that the PDB structures for part of proteins have been updated from 2016 to now. The source codes and experiment data for re-defining DNA-binding sites in 498 protein chains from the PDNA-543 and PDNA-41 datasets are available at <https://github.com/yiheng-zhu/ULDNA/tree/main/Check_TargetDNA>.

**Text S2. Procedures for the ESM2 transformer**

**A. Masking**

For an input sequence, the masking strategy [7] is performed on the corresponding tokens (i.e., amino acids). Specifically, we randomly sample 15% tokens, each of which is changed as a special “masking” token with 80% probability, a randomly chosen alternate amino acid with 10% probability, and the original input token (i.e., no change) with 10% probability.

**B. One-hot encoding**

The masked sequence is represented as a $L\times28$ matrix using one-hot encoding [8], where 28 is the types of tokens, including 20 common amino acids, 6 non-common amino acids (B, J, O, U, X, and Z), 1 gap token, and 1 “masking” token.

**C. Embedding with position information**

The one-hot coding matrix $X$ of the masked sequence is multiplied by an embedding weight matrix $W_{E}$ to generate an embedding matrix $H_{E}$:

$H_{E}=XW_{E}, X\in R^{L\times28}, W_{E}$ $\in R^{28\times D}, H_{E}$ $\in R^{L\times D}$ (S1)

where $L$ is the length of the masked sequence, 28 is the types of tokens in the masked sequence, and $D$ is the embedding dimension.

Then, the position embedding strategy is used to record the position of each token in the masked sequence to generate a position embedding matrix $H_{P}$:

$H_{P}=\left[ \begin{aligned} h_{1} \\ h_{2} \\ \ldots\\ h_{L} \end{aligned} \right], h_{i}=\left( v_{i,1},v_{i,2},{\ldots,v}_{i,D} \right),H_{P}\in R^{L\times D}\text{, and }h_{i}\in R^{D}$ (S2)

$v_{i,2k}=\sin(\frac{i}{{10000}^{2k/D}}){\text{,} v}_{i,2k+1}=\cos(\frac{i}{{10000}^{(2k+1)/D}})$, $k=0, 1, .., (D-1)/2$ (S3)

where$\text{ }h_{i}$ is the embedding vector for the $i$-th position in the masked sequence.

Finally, two embedding matrices are added as a combination embedding matrix $H_{1}:$

$H_{1}=H_{E}+$ $H_{P}, H_{1}$ $\in R^{L\times D}$ (S4)

**D. Self-attention**

The embedding matrix $H_{1}$is fed to a self-attention block with $n$ layers, each of which consists of $m$ attention heads, a linear unit, and a feed-forward network (FFN). In each attention head, the scale dot-product attention is performed as follows:

$A_{i,j}=Softmax(M_{i,j}^{Q}{M_{i,j}^{K}}^{T}/\sqrt{d_{ij}}) M_{i,j}^{V}$ (S5)

$M_{i,j}^{Q}=H_{i}W_{i,j}^{Q}$, $M_{i,j}^{K}=H_{i}W_{i,j}^{K},$ $M_{i,j}^{V}=H_{i}W_{i,j}^{V}$ (S6)

$d_{ij}=D/m$, $W_{i,j}^{Q},W_{i,j}^{K},W_{i,j}^{V}$ $\in R^{D\times(\frac{D}{m})}$, $M_{i,j}^{Q}$, $M_{i,j}^{K}$,$M_{i,j}^{V}\text{,} A_{i,j}\in R^{L\times(\frac{D}{m})}$ (S7)

where $A_{i,j}$ is the attention matrix in the ($i$-th layer, $j$-th head) and measures the evolution correlation for each amino acid pair in the sequence, $M_{i,j}^{Q}$, $M_{i,j}^{K}$, and$M_{i,j}^{V}$are Query, Key, and Value matrices in the ($i$-th layer, $j$-th head), $H_{i}$is the input matrix in the $i$-th layer,$W_{i,j}^{Q}$, $W_{i,j}^{K}$, and $W_{i,j}^{V}$are weight matrices, and $d_{ij}$ is the scale parameter.

The outputs of all attention heads in the $i$-th layer are concatenated as a new matrix $A_{i}$, which is further fed to a linear unit to output the matrix $U_{i}$:

$A_{i}=A_{i,1}A_{i,2}\ldots A_{i,m}$ (S8)

$U_{i}={A_{i}W}_{i}^{1}+b_{i}^{1}, W_{i}^{1}$ $\in R^{D\times D}, A_{i}, b_{i}^{1},U_{i}\in R^{L\times D}$ (S9)

where $W_{i}^{1}$ and $b_{i}^{1}$ are the weight matrix and bias, respectively, in the linear unit.

**E. Feed-forward network with shortcut connections**

The $U_{i}$ is added by $H_{i}$to generate a new matrix $F_{i}$, which is further fed to the FFN to output the matrix $T_{i}$:

$F_{i}$= $H_{i}$ + $U_{i}$ (S10)

$T_{i}=gelu\left( F_{i}W_{i}^{2}+b_{i}^{2} \right)W_{i}^{3}+b_{i}^{3}$, $W_{i}^{2},W_{i}^{3}$ $\in R^{D\times D}$, $b_{i}^{2},b_{i}^{3},T_{i}\in R^{L\times D}$ (S11)

$\mathrm{gelu}\left( x \right)=x\emptyset(x)$ (S12)

where $W_{i}^{2}$ and $W_{i}^{3}$ are weight matrices in the FFN, $b_{i}^{2}$ and $b_{i}^{3}$ are bias in the FFN, and $\emptyset\left( x \right)$is the integral of Gaussian Distribution for $x$

The $F_{i}$ is added by $T_{i}$ as the output of the $i$-th attention layer:

$H_{i+1}$= $F_{i}$+ $T_{i}, H_{i+1}\in R^{L\times D}$ (S13)

where $H_{i+1}$ is the evolution diversity-based embedding matrix in the $i$-th attention layer.

The output of the last attention layer is fed to a fully connected layer with SoftMax function to generate a $L\times28$ probability matrix:

$P=SoftMax\left( H^{n}W^{n}+b^{n} \right), P\in R^{L\times28}$ (S14)

where the ($l$-th, $c$-th) value in $P$ indicates the probability that the $l$-th token in the masked sequence is predicted as the $c$-th type of amino acid, $W^{n}$ and $b^{n}$ are weight matrix and bias, respectively.

**F. Loss function**

The loss function is designed as a negative log-likelihood function between inputted one-hot and outputted probability matrices, to ensure that the prediction model correctly predicts the true amino acids in the masked position as much as possible:

${Loss}_{esm}=E_{x\sim X}\sum_{l\in x(M)} \left( -\frac{logP_{l, c\left( l \right)}}{\left| x\left( M \right) \right|} \right)$ (S15)

where $x$ is a sequence in training protein set $X$, $x(M)$ is a set of masking positions in $x$, $\left| x(M) \right|$ is the number of elements in $x(M)$, $c(l)$ is the type index of amino acid for the $l$-th token in $x$ before masking, and -$logP_{l, c\left( l \right)}$ is the negative log-likelihood of the true amino acid $x_{l}$ under the condition of masking.

The ESM2 transformer is optimized by minimizing the loss function via Adam optimization algorithm [9]. Then, the output of the last attention layer is represented as a $L\times D$ matrix, as the evolution diversity-based embedding for DNA-binding site prediction, where $D$ is the number of neurons of FFN. The current ESM2 model with 3 billion parameters was trained over 60 million proteins from the UniRef50 database and can be freely downloaded at https://github.com/facebookresearch/esm, where $n=36$, $m=20$, and $D=2560$.

**Text S3. Procedures for the ESM-MSA transformer**

**A. Masking**

For an input multiple sequence alignment (MSA), the masking strategy is performed. Specifically, for each individual sequence in MSA, we randomly sample 15% tokens (amino acids), each of which is changed as a special “masking” token with 80% probability, a randomly chosen alternate amino acid with 10% probability, and the original input token (i.e., no change) with 10% probability.

**B. One-hot encoding**

The masked MSA is encoded as three matrices using one-hot encoding from three different views. Specifically, for the $j$-th position of the $i$-th sequence in the masked MSA, we encode it as three one-hot vectors, i.e., $\boldsymbol{x}_{ij}$, $\boldsymbol{y}_{ij}$, and $\boldsymbol{z}_{ij}$, from the views of token type, row position, and column position, respectively.

$\boldsymbol{x}_{ij}=\left( x_{ij1}{, x}_{ij2},\ldots, x_{ijC_{max}} \right)\in R^{C_{max}}, x_{ijk}=\left\{ \begin{aligned} 1, k=c_{ij} \\ 0, k\neq c_{ij} \end{aligned} \right.$ (S16)

$\boldsymbol{y}_{ij}=\left( y_{ij1}{, y}_{ij2},\ldots, y_{ijM_{max}} \right)\in R^{M_{max}}, y_{ijk}=\left\{ \begin{aligned} 1, k=i \\ 0, k\neq i \end{aligned} \right.$ (S17)

$\boldsymbol{z}_{ij}=\left( z_{ij1}{, z}_{ij2},\ldots, z_{ijL_{max}} \right)\in R^{L_{max}}, z_{ijk}=\left\{ \begin{aligned} 1, k=j \\ 0, k\neq j \end{aligned} \right.$ (S18)

where $c_{ij}$ is the index of token type for the $j$-th position of the $i$-th sequence, $C_{max}$ is the number of types of tokens, $L_{max}$ and $M_{max}$ are preset maximum values for sequence length and alignments, respectively. In this work, $C_{max}=28$ and $L_{max}=M_{max}=1024$, where 28 types of tokens include 20 common amino acids, 6 non-common amino acids (B, J, O, U, X, and Z), 1 gap token, and 1 “masking” token.

According to Eqs. S16-S18, the masked MSA can be encoded as three matrices, i.e., $\boldsymbol{X}$, $\boldsymbol{Y}$, and $\boldsymbol{Z}$, through one-hot encoding from the view of token type, row position, and column position, respectively, where $\boldsymbol{X}\in R^{M\times L\times C_{max}}$, $\boldsymbol{Y}\in R^{M\times L\times M_{max}}$, and $\boldsymbol{Z}\in R^{M\times L\times L_{max}}$, $M$ is the number of alignments, and $L$ is the length of individual sequence in the masked MSA.

**C. Initial embedding**

Each one-hot coding matrix is multiplied by a weight matrix to generate the corresponding embedding matrix:

$\boldsymbol{H}_{token}=\boldsymbol{X}\boldsymbol{W}_{token}=\left[ \begin{aligned} \boldsymbol{X}\left[ 1 \right] \\ \boldsymbol{X}\left[ 2 \right] \\ \ldots\\ \boldsymbol{X}\left[ M \right] \end{aligned} \right]\boldsymbol{W}_{token}=\left[ \begin{aligned} \boldsymbol{X}\left[ 1 \right]\boldsymbol{W}_{token} \\ \boldsymbol{X}\left[ 2 \right]\boldsymbol{W}_{token} \\ \ldots\\ \boldsymbol{X}\left[ M \right]\boldsymbol{W}_{token} \end{aligned} \right]\in R^{M\times L\times D}$ (S19)

$$\boldsymbol{X[}i\boldsymbol{]}\in R^{L\times C_{max}}, \boldsymbol{W}_{token}\in R^{C_{max}\times D}$$

$\boldsymbol{H}_{row}=\boldsymbol{X}\boldsymbol{W}_{row}=\left[ \begin{aligned} \boldsymbol{Y}[1] \\ \boldsymbol{Y}[2] \\ \ldots\\ \boldsymbol{Y}[M] \end{aligned} \right]\boldsymbol{W}_{row}=\left[ \begin{aligned} \boldsymbol{Y}[1]\boldsymbol{W}_{row} \\ \boldsymbol{Y}[2]\boldsymbol{W}_{row} \\ \ldots\\ \boldsymbol{Y}[M]\boldsymbol{W}_{row} \end{aligned} \right]\in R^{M\times L\times D}$ (S20)

$$\boldsymbol{Y}[i]\in R^{L\times M_{max}}, \boldsymbol{W}_{row}\in R^{M_{max}\times D}$$

$\boldsymbol{H}_{col}=\boldsymbol{Z}\boldsymbol{W}_{col}=\left[ \begin{aligned} \boldsymbol{Z}[1] \\ \boldsymbol{Z}[2] \\ \ldots\\ \boldsymbol{Z}[M] \end{aligned} \right]\boldsymbol{W}_{col}=\left[ \begin{aligned} \boldsymbol{Z}[1]\boldsymbol{W}_{col} \\ \boldsymbol{Z}[2]\boldsymbol{W}_{col} \\ \ldots\\ \boldsymbol{Z}[M]\boldsymbol{W}_{col} \end{aligned} \right]\in R^{M\times L\times D}$ (S21)

$$\boldsymbol{Z}[i]\in R^{L\times L_{max}}, \boldsymbol{W}_{col}\in R^{L_{max}\times D}$$

where $\boldsymbol{X[}i\boldsymbol{]}$, $\boldsymbol{Y[}i\boldsymbol{]}$ and $\boldsymbol{Z[}i\boldsymbol{]}$ are the one-hot coding matrices for the $i$-th sequence in the masked MSA from the view of token type, row position, and column position, respectively, $\boldsymbol{H}_{token}$, $\boldsymbol{H}_{row}$, and $\boldsymbol{H}_{col}$ are token type-based, row position-based, and column position-based embedding matrices for the masked MSA, respectively, and $D$ is the embedding dimension. In this work, $D=768$.

Three embedding matrices are added as an initial embedding matrix $\boldsymbol{H}_{init}$:

$\boldsymbol{H}_{init}=\boldsymbol{H}_{token}+\boldsymbol{H}_{row}+\boldsymbol{H}_{col}, \boldsymbol{H}_{init}\in R^{M\times L\times D}$ (S22)

**D. Batch normalization and dropout**

The initial embedding matrix $\boldsymbol{H}_{init}$ is fed to the batch normalization layer to generate the corresponding normalized matrix $\boldsymbol{H}_{1}$:

$\boldsymbol{H}_{1}=BN(\boldsymbol{H}_{init})=\left[ \begin{matrix} BN(\boldsymbol{h}_{11}) & \cdots& BN(\boldsymbol{h}_{1L}) \\ \vdots& \ddots& \vdots\\ BN(\boldsymbol{h}_{M1}) & \cdots& BN(\boldsymbol{h}_{ML}) \end{matrix} \right]$ (S23)

$BN\left( \boldsymbol{h}_{ij} \right)=\gamma\cdot\frac{\boldsymbol{h}_{ij}-u_{ij}}{\sqrt{\sigma_{ij}^{2}+\epsilon}}+\beta,\boldsymbol{h}_{ij}\in R^{D}$ (S24)

where $\boldsymbol{h}_{ij}$ is the initial embedding vector for the $j$-th position of the $i$-th sequence in the masked MSA, $u_{ij}$ and $\sigma_{ij}^{2}$ are mean and variance for $\boldsymbol{h}_{ij}$, respectively, and $\gamma$, $\beta$, and $\epsilon$ are normalized factors.

The normalized matrix $\boldsymbol{H}_{1}$ is fed to the dropout layer:

$\boldsymbol{H}_{1}\leftarrow dropout(\boldsymbol{H}_{1}, r)$ (S25)

where $r$ is the rate of neurons that are randomly dropped in each training step, indicating that the corresponding weight vectors will be not optimized.

**E. Self-attention**

The initial embedding matrix $\boldsymbol{H}_{1}$ is fed to the self-attention network with $N$ blocks, each of which consists of three sub-blocks. In this work, $N=12$.

The first sub-block consists of a batch normalization layer, a row attention layer, a dropout layer, and a short connection.

$\boldsymbol{H}_{k}^{B}=BN(\boldsymbol{H}_{k})$ (S26)

$\boldsymbol{H}_{k}^{R}=RA(\boldsymbol{H}_{k}^{B})$ (S27)

$\boldsymbol{H}_{k}^{R}\leftarrow dropout(\boldsymbol{H}_{k}^{R}, r)$ (S28)

$\boldsymbol{F}_{k}=SC\left( \boldsymbol{H}_{k},\boldsymbol{H}_{k}^{R} \right)=\boldsymbol{H}_{k}+\boldsymbol{H}_{k}^{R}$ (S29)

where $\boldsymbol{H}_{k}$ and $\boldsymbol{F}_{k}$ are the input and output matrices in the first sub-block of the $k$-th self-attention block, respectively, $BN(\cdot)$ is the batch normalization function (see Eqs. S23-S24), $SC(\cdot)$ is the short connection, and $RA(\cdot)$ is the row attention layer (see Eqs. S38-S45), $\boldsymbol{H}_{k}$, $\boldsymbol{H}_{k}^{B}$, $\boldsymbol{H}_{k}^{R}$, $\boldsymbol{F}_{k}\in R^{M\times L\times D}$.

The second sub-block consists of a batch normalization layer, a column attention layer, a dropout layer, and a short connection.

$\boldsymbol{F}_{k}^{B}=BN(\boldsymbol{F}_{k})$ (S30)

$\boldsymbol{F}_{k}^{C}=CA(\boldsymbol{F}_{k}^{B})$ (S31)

$\boldsymbol{F}_{k}^{C}\leftarrow dropout(\boldsymbol{F}_{k}^{C}, r)$ (S32)

$\boldsymbol{U}_{k}=SC\left( \boldsymbol{F}_{k},\boldsymbol{F}_{k}^{C} \right)=\boldsymbol{F}_{k}+\boldsymbol{F}_{k}^{C}$ (S33)

where $\boldsymbol{F}_{k}$ and $\boldsymbol{U}_{k}$ are the input and output matrices in the second sub-block of the $k$-th self-attention block, respectively, $CA(\cdot)$ is the column attention layer (see Eqs. S46-S54), and $\boldsymbol{F}_{k}^{B}$, $\boldsymbol{F}_{k}^{C}$, $\boldsymbol{U}_{k}\in R^{M\times L\times D}$.

The last sub-block consists of a batch normalization layer, a feed-forward network, a dropout layer, and a short connection.

$\boldsymbol{U}_{k}^{B}=BN(\boldsymbol{U}_{k})$ (S34)

$\boldsymbol{U}_{k}^{F}=FFN(\boldsymbol{U}_{k}^{B})$ (S35)

$\boldsymbol{U}_{k}^{F}\leftarrow dropout(\boldsymbol{U}_{k}^{F}, r)$ (S36)

$\boldsymbol{H}_{k+1}=SC\left( \boldsymbol{U}_{k},\boldsymbol{U}_{k}^{F} \right)=\boldsymbol{U}_{k}+\boldsymbol{U}_{k}^{F}$ (S37)

where $\boldsymbol{U}_{k}$ and $\boldsymbol{H}_{k+1}$ are the input and output matrices in the third sub-block of the $k$-th self-attention block, respectively, $FFN(.)$ is the feed-forward network (see Eqs. S55-S60), and $\boldsymbol{U}_{k}^{B}$, $\boldsymbol{U}_{k}^{F}$, $\boldsymbol{H}_{k+1}\in R^{M\times L\times D}$.

**(a) Row attention**

Each row attention layer consists of $m$ attention heads and a linear unit, where $m=12$. In each attention head, the input matrix is multiplied by three weight matrices to generate the corresponding Query, Key, and Value matrices.

$\boldsymbol{Q}_{kt}^{R}=\boldsymbol{H}_{k}^{B}\boldsymbol{W}_{kt}^{QR}=\left[ \begin{aligned} \boldsymbol{H}_{k}^{B}[1] \\ \boldsymbol{H}_{k}^{B}[2] \\ \ldots\\ \boldsymbol{H}_{k}^{B}[M] \end{aligned} \right]\boldsymbol{W}_{kt}^{QR}=\left[ \begin{aligned} \boldsymbol{H}_{k}^{B}[1]\boldsymbol{W}_{kt}^{QR} \\ \boldsymbol{H}_{k}^{B}[2]\boldsymbol{W}_{kt}^{QR} \\ \ldots\\ \boldsymbol{H}_{k}^{B}[M]\boldsymbol{W}_{kt}^{QR} \end{aligned} \right]\in R^{M\times L\times(\frac{D}{m})}$ (S38)

$\boldsymbol{K}_{kt}^{R}=\boldsymbol{H}_{k}^{B}\boldsymbol{W}_{kt}^{KR}=\left[ \begin{aligned} \boldsymbol{H}_{k}^{B}[1] \\ \boldsymbol{H}_{k}^{B}[2] \\ \ldots\\ \boldsymbol{H}_{k}^{B}[M] \end{aligned} \right]\boldsymbol{W}_{kt}^{KR}=\left[ \begin{aligned} \boldsymbol{H}_{k}^{B}[1]\boldsymbol{W}_{kt}^{KR} \\ \boldsymbol{H}_{k}^{B}[2]\boldsymbol{W}_{kt}^{KR} \\ \ldots\\ \boldsymbol{H}_{k}^{B}[M]\boldsymbol{W}_{kt}^{KR} \end{aligned} \right]\in R^{M\times L\times(\frac{D}{m})}$ (S39)

$\boldsymbol{V}_{kt}^{R}=\boldsymbol{H}_{k}^{B}\boldsymbol{W}_{kt}^{VR}=\left[ \begin{aligned} \boldsymbol{H}_{k}^{B}[1] \\ \boldsymbol{H}_{k}^{B}[2] \\ \ldots\\ \boldsymbol{H}_{k}^{B}[M] \end{aligned} \right]\boldsymbol{W}_{kt}^{VR}=\left[ \begin{aligned} \boldsymbol{H}_{k}^{B}[1]\boldsymbol{W}_{kt}^{VR} \\ \boldsymbol{H}_{k}^{B}[2]\boldsymbol{W}_{kt}^{VR} \\ \ldots\\ \boldsymbol{H}_{k}^{B}[M]\boldsymbol{W}_{kt}^{VR} \end{aligned} \right]\in R^{M\times L\times(\frac{D}{m})}$ (S40)

$$\boldsymbol{H}_{k}^{B}\left[ i \right]\in R^{L\times D}, \boldsymbol{W}_{kt}^{QR},\boldsymbol{W}_{kt}^{KR},\boldsymbol{W}_{kt}^{VR}\in R^{D\times(\frac{D}{m})}$$

where $\boldsymbol{H}_{k}^{B}$ is the input matrix of row attention layer in the $k$-th self-attention block (See Eq. S27), $\boldsymbol{Q}_{kt}^{R}$, $\boldsymbol{K}_{kt}^{R}$, and $\boldsymbol{V}_{kt}^{R}$ are Query, Key, and Value matrices in the *t*-th head of the row attention layer in the $k$-th block, respectively, $\boldsymbol{W}_{kt}^{QR}$, $\boldsymbol{W}_{kt}^{KR}$, and $\boldsymbol{W}_{kt}^{VR}$ are corresponding weight matrices.

Then, the dot-product between $\boldsymbol{Q}_{kt}^{R}$ and $\boldsymbol{K}_{kt}^{R}$ is performed and then normalized by SoftMax function to generate a row attention weight matrix:

$\boldsymbol{W}_{kt}^{AR}=SoftMax(\frac{\sum_{i=1}^{M} \boldsymbol{Q}_{kt}^{R}[i]\cdot{(\boldsymbol{K}_{kt}^{R}[i])}^{T}\}}{\sqrt{MD/m}})\in R^{L\times L},$ $\boldsymbol{Q}_{kt}^{R}[i]$, $\boldsymbol{K}_{kt}^{R}[i] \in R^{L\times(D/m)}$ (S41)

$\boldsymbol{W}_{kt}^{AR}\leftarrow dropout(\boldsymbol{W}_{kt}^{AR}, r)$ (S42)

where $\boldsymbol{W}_{kt}^{AR}$ is the attention weight matrix in the *t*-th head of the row attention layer in the $k$-th block and measures the correlation for each pair of columns in the masked MSA.

Next, the row attention weight matrix $\boldsymbol{W}_{kt}^{AR}$ is multiplied by the Value matrix $\boldsymbol{V}_{kt}^{R}$ to generate the corresponding row attention matrix:

$\boldsymbol{A}_{kt}^{R}=\boldsymbol{W}_{kt}^{AR}\boldsymbol{V}_{kt}^{R}=\boldsymbol{W}_{kt}^{AR}\left[ \begin{aligned} \boldsymbol{V}_{kt}^{R}[1] \\ \boldsymbol{V}_{kt}^{R}[2] \\ \ldots\\ \boldsymbol{V}_{kt}^{R}[M] \end{aligned} \right]=\left[ \begin{aligned} \boldsymbol{W}_{kt}^{AR}\boldsymbol{V}_{kt}^{R}[1] \\ \boldsymbol{W}_{kt}^{AR}\boldsymbol{V}_{kt}^{R}[2] \\ \ldots\\ {\boldsymbol{W}_{kt}^{AR}\boldsymbol{V}}_{kt}^{R}[M] \end{aligned} \right]\in R^{M\times L\times\left( \frac{D}{m} \right)},\boldsymbol{V}_{kt}^{R}\left[ i \right]\in R^{L\times\left( \frac{D}{m} \right)}$ (S43)

where $\boldsymbol{A}_{kt}^{R}$ is the attention matrix in the *t*-th head of the row attention layer in the $k$-th block.

Finally, the outputs of all attention heads are concatenated as a new matrix, which is further fed to a linear unit:

$\boldsymbol{A}_{k}^{R}=\boldsymbol{A}_{k1}^{R}\boldsymbol{A}_{k2}^{R}\ldots\boldsymbol{A}_{km}^{R}\in R^{M\times L\times D}$ (S44)

$\boldsymbol{H}_{k}^{R}=\boldsymbol{A}_{k}^{R}\boldsymbol{W}_{k}^{R}+\boldsymbol{b}_{k}^{R}=\left[ \begin{aligned} \boldsymbol{A}_{k}^{R}[1] \\ \boldsymbol{A}_{k}^{R}[2] \\ \ldots\\ \boldsymbol{A}_{k}^{R}[M] \end{aligned} \right]\boldsymbol{W}_{k}^{R}+\boldsymbol{b}_{k}^{R}=\left[ \begin{aligned} \boldsymbol{A}_{k}^{R}[1]\boldsymbol{W}_{k}^{R} \\ \boldsymbol{A}_{k}^{R}[2]\boldsymbol{W}_{k}^{R} \\ \ldots\\ \boldsymbol{A}_{k}^{R}[M]\boldsymbol{W}_{k}^{R} \end{aligned} \right]+\boldsymbol{b}_{k}^{R}\in R^{M\times L\times D}$ (S45)

$\boldsymbol{W}_{k}^{R}\in R^{D\times D},\boldsymbol{A}_{k}^{R}\left[ i \right]\in R^{L\times D}$

where $\boldsymbol{H}_{k}^{R}$ in the output matrix of row attention layer in the $k$-th attention block (See Eq. S27), and $\boldsymbol{W}_{k}^{R}$ and $\boldsymbol{b}_{k}^{R}$ are weight matrix and bias in the linear unit, respectively.

**(b) Column attention**

Each column attention layer consists of $m$ attention heads and a linear unit. In each attention head, the input matrix is multiplied by three weight matrices to generate the corresponding Query, Key, and Value matrices.

$\boldsymbol{Q}_{kt}^{C}=\boldsymbol{F}_{k}^{B}\boldsymbol{W}_{kt}^{QC}=\left[ \begin{aligned} \boldsymbol{F}_{k}^{B}[1] \\ \boldsymbol{F}_{k}^{B}[2] \\ \ldots\\ \boldsymbol{F}_{k}^{B}[M] \end{aligned} \right]\boldsymbol{W}_{kt}^{QC}=\left[ \begin{aligned} \boldsymbol{F}_{k}^{B}[1]\boldsymbol{W}_{kt}^{QC} \\ \boldsymbol{F}_{k}^{B}[2]\boldsymbol{W}_{kt}^{QC} \\ \ldots\\ \boldsymbol{F}_{k}^{B}[M]\boldsymbol{W}_{kt}^{QC} \end{aligned} \right]\in R^{M\times L\times(\frac{D}{m})}$ (S46)

$\boldsymbol{K}_{kt}^{C}=\boldsymbol{F}_{k}^{B}\boldsymbol{W}_{kt}^{KC}=\left[ \begin{aligned} \boldsymbol{F}_{k}^{B}[1] \\ \boldsymbol{F}_{k}^{B}[2] \\ \ldots\\ \boldsymbol{F}_{k}^{B}[M] \end{aligned} \right]\boldsymbol{W}_{kt}^{KC}=\left[ \begin{aligned} \boldsymbol{F}_{k}^{B}[1]\boldsymbol{W}_{kt}^{KC} \\ \boldsymbol{F}_{k}^{B}[2]\boldsymbol{W}_{kt}^{KC} \\ \ldots\\ \boldsymbol{F}_{k}^{B}[M]\boldsymbol{W}_{kt}^{KC} \end{aligned} \right]\in R^{M\times L\times(\frac{D}{m})}$ (S47)

$\boldsymbol{V}_{kt}^{C}=\boldsymbol{F}_{k}^{B}\boldsymbol{W}_{kt}^{VC}=\left[ \begin{aligned} \boldsymbol{F}_{k}^{B}[1] \\ \boldsymbol{F}_{k}^{B}[2] \\ \ldots\\ \boldsymbol{F}_{k}^{B}[M] \end{aligned} \right]\boldsymbol{W}_{kt}^{VC}=\left[ \begin{aligned} \boldsymbol{F}_{k}^{B}[1]\boldsymbol{W}_{kt}^{VC} \\ \boldsymbol{F}_{k}^{B}[2]\boldsymbol{W}_{kt}^{VC} \\ \ldots\\ \boldsymbol{F}_{k}^{B}[M]\boldsymbol{W}_{kt}^{VC} \end{aligned} \right]\in R^{M\times L\times(\frac{D}{m})}$ (S48)

$$\boldsymbol{F}_{k}^{B}\left[ i \right]\in R^{L\times D}, \boldsymbol{W}_{kt}^{QC}\boldsymbol{, W}_{kt}^{KC}, \boldsymbol{W}_{kt}^{VC}\in R^{D\times(\frac{D}{m})}$$

where $\boldsymbol{F}_{k}^{B}$ is the input matrix of column attention layer in the $k$-th self-attention block (see Eq. S31), $\boldsymbol{Q}_{kt}^{C}$, $\boldsymbol{K}_{kt}^{C}$, and $\boldsymbol{V}_{kt}^{C}$ are Query, Key, and Value matrices in the *t*-th head of column attention layer in the $k$-th block, respectively, $\boldsymbol{W}_{kt}^{QC}$, $\boldsymbol{W}_{kt}^{KC}$, and $\boldsymbol{W}_{kt}^{VC}$ are corresponding weight metrices.

Then, the dot-product between $\boldsymbol{Q}_{kt}^{C}$ and $\boldsymbol{K}_{kt}^{C}$ is performed and then normalized by SoftMax function to generate an attention weight matrix:

$\boldsymbol{W}_{kt}^{AC}=SoftMax\left( \frac{\boldsymbol{Q}_{kt}^{C}{\boldsymbol{(K}_{kt}^{C})}^{T}}{\sqrt{D/m}} \right)\in R^{M\times L\times M}$ (S49)

$\boldsymbol{W}_{kt}^{AC}\leftarrow dropout(\boldsymbol{W}_{kt}^{AC}, r)$ (S50)

$\boldsymbol{Q}_{kt}^{C}{\boldsymbol{(K}_{kt}^{C})}^{T}=\left[ \boldsymbol{Q}_{kt}^{C}\left[ :, 1,: \right] \boldsymbol{Q}_{kt}^{C}\left[ :, 2, : \right] \boldsymbol{\ldots Q}_{kt}^{C}\left[ :, L, : \right] \right]{\cdot\left[ \boldsymbol{K}_{kt}^{C}\left[ :, 1,: \right] \boldsymbol{K}_{kt}^{C}\left[ :, 2, : \right] \boldsymbol{\ldots K}_{kt}^{C}\left[ :, L, : \right] \right]}^{T}=\left[ \boldsymbol{Q}_{kt}^{C}\left[ :, 1,: \right]{\boldsymbol{\cdot K}_{kt}^{C}\left[ :, 1,: \right]}^{T} \boldsymbol{Q}_{kt}^{C}\left[ :, 2,: \right]{\boldsymbol{\cdot K}_{kt}^{C}\left[ :, 2,: \right]}^{T}\ldots\boldsymbol{Q}_{kt}^{C}\left[ :, L,: \right]{\boldsymbol{\cdot K}_{kt}^{C}\left[ :, L,: \right]}^{T} \right]\in R^{M\times L\times M}$ (S51)

$$\boldsymbol{Q}_{kt}^{C}\left[ :, j,: \right], \boldsymbol{K}_{kt}^{C}\left[ :, j,: \right]\in R^{M\times\left( \frac{D}{m} \right)}, \boldsymbol{Q}_{kt}^{C}\left[ :, j,: \right]{\boldsymbol{\cdot K}_{kt}^{C}\left[ :, j,: \right]}^{T}\in R^{M\times M}$$

where $\boldsymbol{W}_{kt}^{AC}$ is the attention weight matrix in the *t*-th head of column attention layer in the $k$-th block, and $\boldsymbol{W}_{kt}^{AC}\left[ :, j,: \right]$ measures the correlation for each pair of alignments at the $j$-th position.

Next, the column attention weight matrix $\boldsymbol{W}_{kt}^{AC}$ is multiplied by Value matrix $\boldsymbol{V}_{kt}^{C}$ to generate the corresponding column attention matrix:

$\boldsymbol{A}_{kt}^{C}=\boldsymbol{W}_{kt}^{AC}\boldsymbol{V}_{kt}^{C}=\left[ \boldsymbol{W}_{kt}^{AC}\left[ :, 1,: \right] \boldsymbol{W}_{kt}^{AC}\left[ :, 2, : \right] \boldsymbol{\ldots W}_{kt}^{AC}\left[ :, L, : \right] \right]\cdot\left[ \boldsymbol{V}_{kt}^{C}\left[ :, 1,: \right] \boldsymbol{V}_{kt}^{C}\left[ :, 2, : \right] \boldsymbol{\ldots V}_{kt}^{C}\left[ :, L, : \right] \right]$=$\left[ \boldsymbol{W}_{kt}^{AC}\left[ :, 1,: \right]\boldsymbol{\cdot V}_{kt}^{C}\left[ :, 1,: \right] \boldsymbol{W}_{kt}^{AC}\left[ :, 2, : \right]\cdot\boldsymbol{V}_{kt}^{C}\left[ :, 2, : \right] \boldsymbol{\ldots W}_{kt}^{AC}\left[ :, L, : \right]\cdot\boldsymbol{V}_{kt}^{C}\left[ :, L, : \right] \right]{\in R}^{M\times L\times(\frac{D}{m})}$ (S52)

$$\boldsymbol{W}_{kt}^{AC}\left[ :, j,: \right]{\in R}^{M\times M}, \boldsymbol{V}_{kt}^{C}\left[ :, j, : \right]{\in R}^{M\times(\frac{D}{m})}, \boldsymbol{W}_{kt}^{AC}\left[ :, j, : \right]\cdot\boldsymbol{V}_{kt}^{C}\left[ :, j, : \right]{\in R}^{M\times(\frac{D}{m})}$$

where $\boldsymbol{A}_{kt}^{C}$ is the attention matrix in the *t*-th head of column attention layer in the $k$-th block.

Finally, the outputs of all attention heads are concatenated as a new matrix, which is further fed to a linear unit:

$\boldsymbol{A}_{k}^{C}=\boldsymbol{A}_{k1}^{C}\boldsymbol{A}_{k2}^{C}\ldots\boldsymbol{A}_{km}^{C}\in R^{M\times L\times D}$ (S53)

$\boldsymbol{F}_{k}^{C}=\boldsymbol{A}_{k}^{C}\boldsymbol{W}_{k}^{C}+\boldsymbol{b}_{k}^{C}=\left[ \begin{aligned} \boldsymbol{A}_{k}^{C}[1] \\ \boldsymbol{A}_{k}^{C}[2] \\ \ldots\\ \boldsymbol{A}_{k}^{C}[M] \end{aligned} \right]\boldsymbol{W}_{k}^{C}=\left[ \begin{aligned} \boldsymbol{A}_{1}^{C}[1]\boldsymbol{W}_{k}^{C} \\ \boldsymbol{A}_{2}^{C}[2]\boldsymbol{W}_{k}^{C} \\ \ldots\\ \boldsymbol{A}_{k}^{C}[M]\boldsymbol{W}_{k}^{C} \end{aligned} \right]+\boldsymbol{b}_{k}^{C}\in R^{M\times L\times D}$ (S54)

$\boldsymbol{W}_{k}^{C}\in R^{D\times D},\boldsymbol{A}_{k}^{C}\left[ i \right]\in R^{L\times D}$

where $\boldsymbol{F}_{k}^{C}$ in the output matrix of column attention layer in the $k$-th attention block, (See Eq. S31), and $\boldsymbol{W}_{k}^{C}$ and $\boldsymbol{b}_{k}^{C}$ are weight matrix and bias in the linear unit, respectively.

**(c) Feed-forward network**

$\boldsymbol{T}_{k}^{F}={gelu(\boldsymbol{U}}_{k}^{B}\boldsymbol{W}_{k}^{1}+\boldsymbol{b}_{k}^{1})\in R^{M\times L\times D_{1}}$ (S55)

$\boldsymbol{T}_{k}^{F}\leftarrow dropout(\boldsymbol{T}_{k}^{F}, r)$ (S56)

$\boldsymbol{U}_{k}^{F}=\boldsymbol{T}_{k}^{F}\boldsymbol{W}_{k}^{2}+\boldsymbol{b}_{k}^{2}\in R^{M\times L\times D}$ (S57)

$gelu\left( x \right)=x\emptyset(x)$ (S58)

$\boldsymbol{U}_{k}^{B}\boldsymbol{W}_{k}^{1}=\left[ \begin{aligned} \boldsymbol{U}_{k}^{B}[1] \\ \boldsymbol{U}_{k}^{B}[2] \\ \ldots\\ \boldsymbol{U}_{k}^{B}[M] \end{aligned} \right]\boldsymbol{W}_{k}^{1}=\left[ \begin{aligned} \boldsymbol{U}_{k}^{B}[1]\boldsymbol{W}_{k}^{1} \\ \boldsymbol{U}_{k}^{B}[2]\boldsymbol{W}_{k}^{1} \\ \ldots\\ \boldsymbol{U}_{k}^{B}[M]\boldsymbol{W}_{k}^{1} \end{aligned} \right]\in R^{M\times L\times D_{1}}$ (S59)

$\boldsymbol{T}_{k}^{F}\boldsymbol{W}_{k}^{2}=\left[ \begin{aligned} \boldsymbol{T}_{k}^{F}[1] \\ \boldsymbol{T}_{k}^{F}[2] \\ \ldots\\ \boldsymbol{T}_{k}^{F}[M] \end{aligned} \right]\boldsymbol{W}_{k}^{2}=\left[ \begin{aligned} \boldsymbol{T}_{k}^{F}[1]\boldsymbol{W}_{k}^{2} \\ \boldsymbol{T}_{k}^{F}[2]\boldsymbol{W}_{k}^{2} \\ \ldots\\ \boldsymbol{T}_{k}^{F}[M]\boldsymbol{W}_{k}^{2} \end{aligned} \right]\in R^{M\times L\times D}$ (S60)

$\boldsymbol{U}_{k}^{B}\left[ i \right]\in R^{L\times D},\boldsymbol{W}_{k}^{1}\in R^{D\times D_{1}}, {\boldsymbol{T}_{k}^{F}\left[ i \right]\in R^{L\times D_{1}}\boldsymbol{,W}}_{k}^{2}\in R^{D_{1}\times D}, D_{1}$=3072

where $\boldsymbol{U}_{k}^{B}$ and $\boldsymbol{U}_{k}^{F}$ are the input and output matrices of the feed-forward network in the $k$-th self-attention block, respectively, (see Eq. S35), $\boldsymbol{W}_{k}^{1}$ and $\boldsymbol{W}_{k}^{2}$ are weight matrices, $\boldsymbol{b}_{k}^{1}$ and $\boldsymbol{b}_{k}^{2}$ are bias, and $\emptyset\left( x \right)$is the integral of Gaussian Distribution for $x$.

**F. Output layer**

The output of the last self-attention block is fed to a fully connected layer with SoftMax function to generate a probability matrix:

$\boldsymbol{P}=SoftMax\left( \boldsymbol{H}_{N+1}\boldsymbol{W}^{O}+\boldsymbol{b}^{O} \right)\in R^{M\times L\times C_{max}}$ (S61)

$\boldsymbol{H}_{N+1}\boldsymbol{W}^{O}=\left[ \begin{aligned} \boldsymbol{H}_{N+1}[1]\boldsymbol{W}^{O} \\ \boldsymbol{H}_{N+1}[2]\boldsymbol{W}^{O} \\ \ldots\\ \boldsymbol{H}_{N+1}[M]\boldsymbol{W}^{O} \end{aligned} \right], \boldsymbol{H}_{N+1}[i]\in R^{L\times D}, \boldsymbol{W}^{O}\in R^{D\times C_{max}}$ (S62)

where $\boldsymbol{H}_{N+1}$ is the outputted embedding matrix in the $N$-th self-attention block, $\boldsymbol{W}^{O}$ and $\boldsymbol{b}^{O}$ are weight matrix and bias, respectively, and the $\boldsymbol{P}(i,j,c)$ indicates the probability that the $j$-th position of the $i$-th sequence in the masked MSA is predicted as the $c$-th type of amino acid.

**G. Loss function**

For an individual MSA, the loss function is designed as:

${Loss}_{msa}=\frac{1}{M}\cdot\sum_{i=1}^{M} \{\frac{1}{\left| mask\left( i \right) \right|}\cdot\sum_{j\in mask\left( i \right)} -log\boldsymbol{P}_{i,j, c(i,j)}\}$ (S63)

where $M$ is the number of alignments, $mask(i)$ is a set of masking positions in the $i$-th sequence, $\left| mask(i) \right|$ is the number of elements in $mask(i)$, $c(i,j)$ is the type index of amino acid for the $j$-th position in the $i$-th sequence before masking, and -$log\boldsymbol{P}_{i,j, c(i,j)}$ is the negative log-likelihood of the true amino acid at the $j$-th position in the $i$-th sequence under the condition of masking.

**Text S4. *p*-value calculation between EPE and the other six feature embeddings**

We select the two-sided Student’s t-test [10] to calculate the *p*-values between EPE and the other six feature embeddings. Specifically, for each feature embedding, we fed it to the designed LSTM-attention network to train a DNA-binding site prediction model, which is then evaluated on the test dataset to calculate the corresponding evaluation index, such as MCC and AUROC values. To reduce the influence of randomness, we repeat this procedure 10 times to generate a group of 10 evaluation indices. Finally, the *p*-value between two feature embeddings is calculated on their groups of evaluation indices under the two-sided Student’s t-test. In this work, we use the Python package “scipy” to implement the Student's t-test to calculate the *p*-values.

**Text S5.** **Procedures for constructing PDNA-960 and PDNA-136 datasets**

Firstly, we downloaded 7345 protein-DNA complex structures which were released in the PDB database before October 15, 2023. In each complex structure, we removed the protein chains whose lengths are more than 1000 or less than 30. Then, the CD-HIT software [11] with a cut-off of 30% sequence identity was performed on all protein chains to remove the redundant chains. After this, we collected 1096 non-redundant protein chains, each of which was labeled with DNA-binding sites using the criteria of Critical Assessment of Structure Prediction (CASP) [2, 3] (see details in Text S1). Finally, the 136 chains released in the PDB after January 1, 2023, were used as the test dataset (i.e., PDNA-136, 2193 DNA-binding sites, and 47287 non-DNA-binding sites), while the remaining 960 chains were used as the training dataset (i.e., PDNA-960, 18336 DNA-bindings sites, and 271988 non-DNA-binding sites).

**Text S6. Formulas for calculating average precision**

In the test dataset, the average precision is calculated as follows:

$AP=\frac{1}{n}\cdot\sum_{i=1}^{n} \frac{{TP}_{i}}{{TP}_{i}+{FP}_{i}}$ (S64)

where ${TP}_{i}$ and ${FP}_{i}$ are the numbers of true positives and false positives, respectively, in the *i*-th test protein, and $n$ is the number of test proteins.

**Supporting Figures**

**
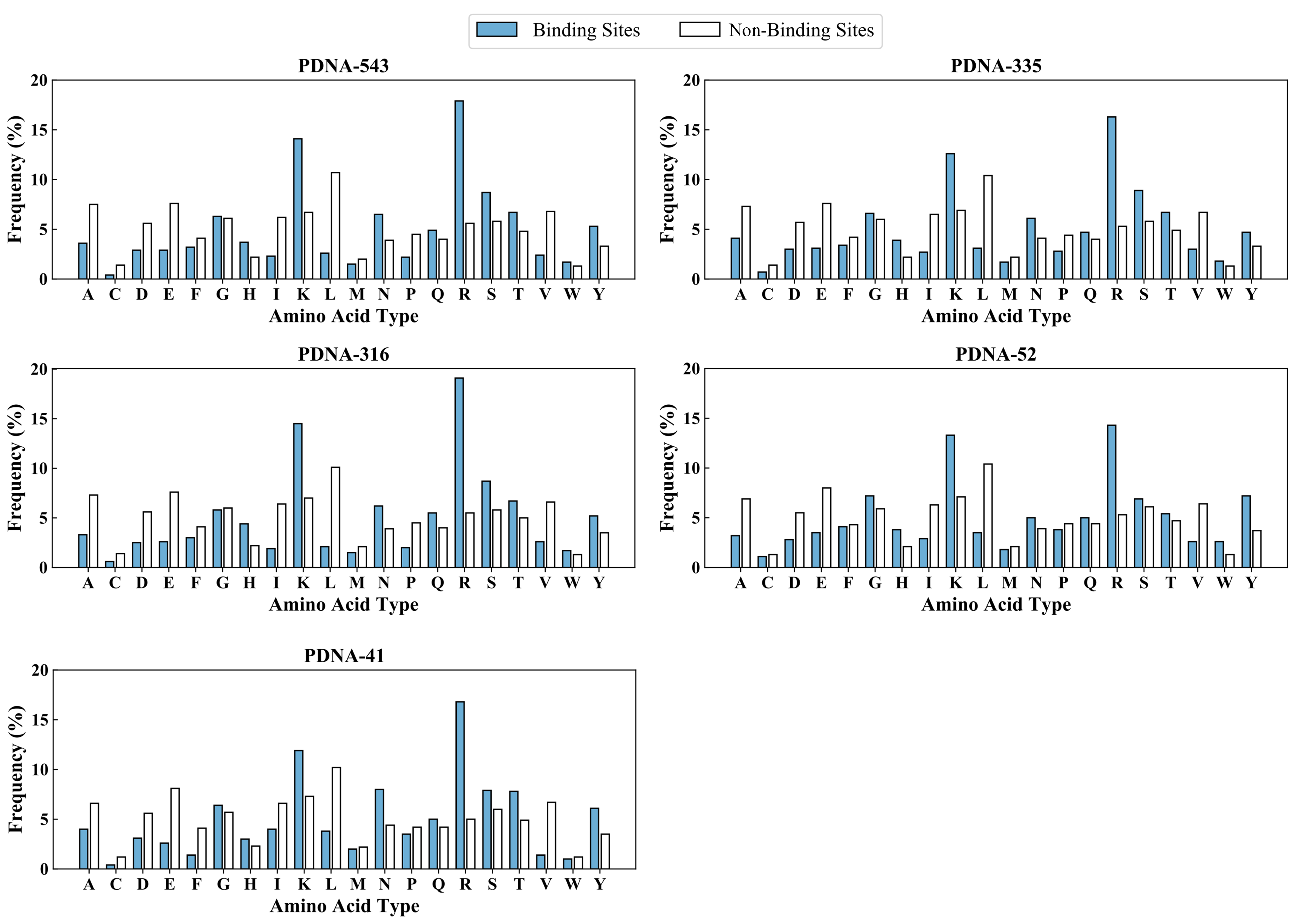
**

Figure S1. The frequency distributions of 20 native amino acids among DNA-binding and non-DNA-binding sites for five benchmark datasets.


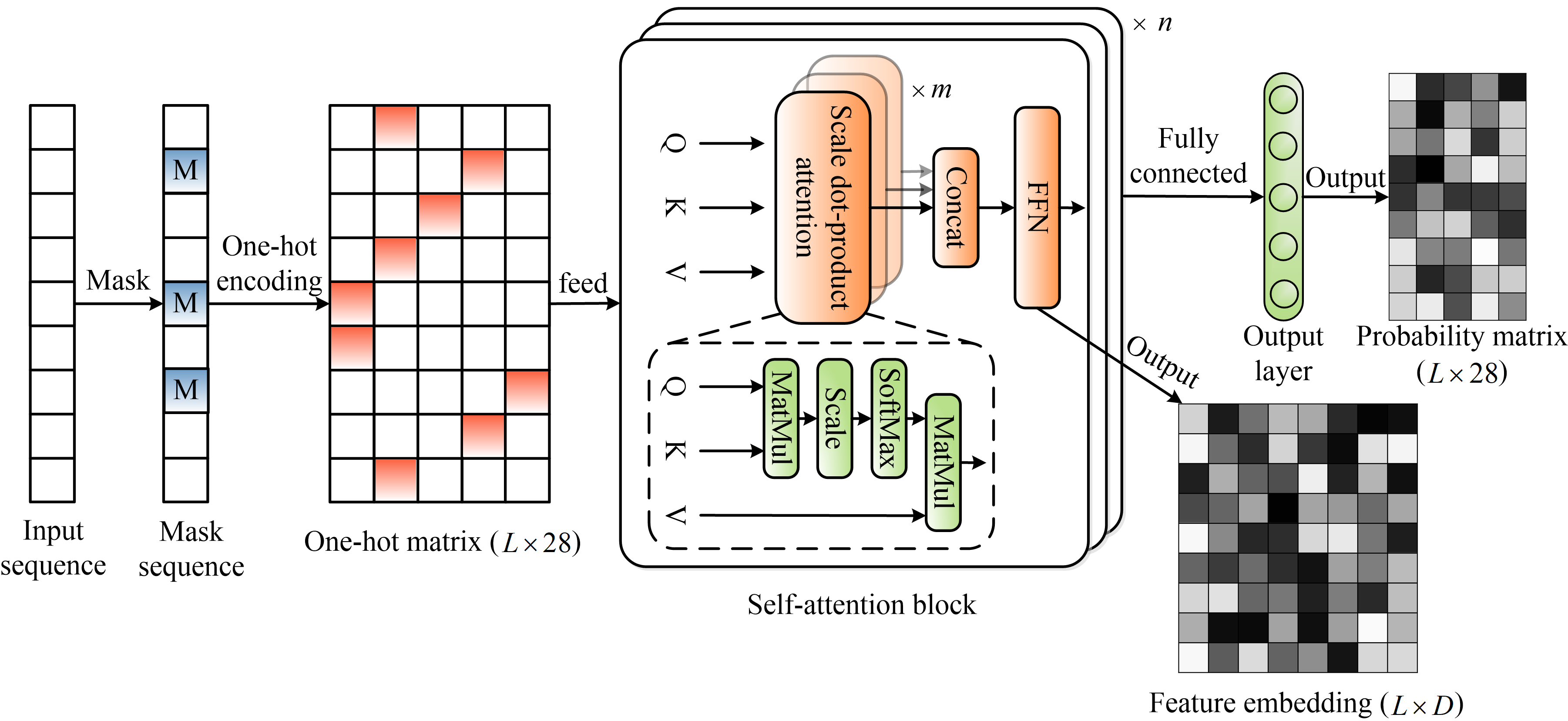


Figure S2. The workflow of the ESM2 transformer.


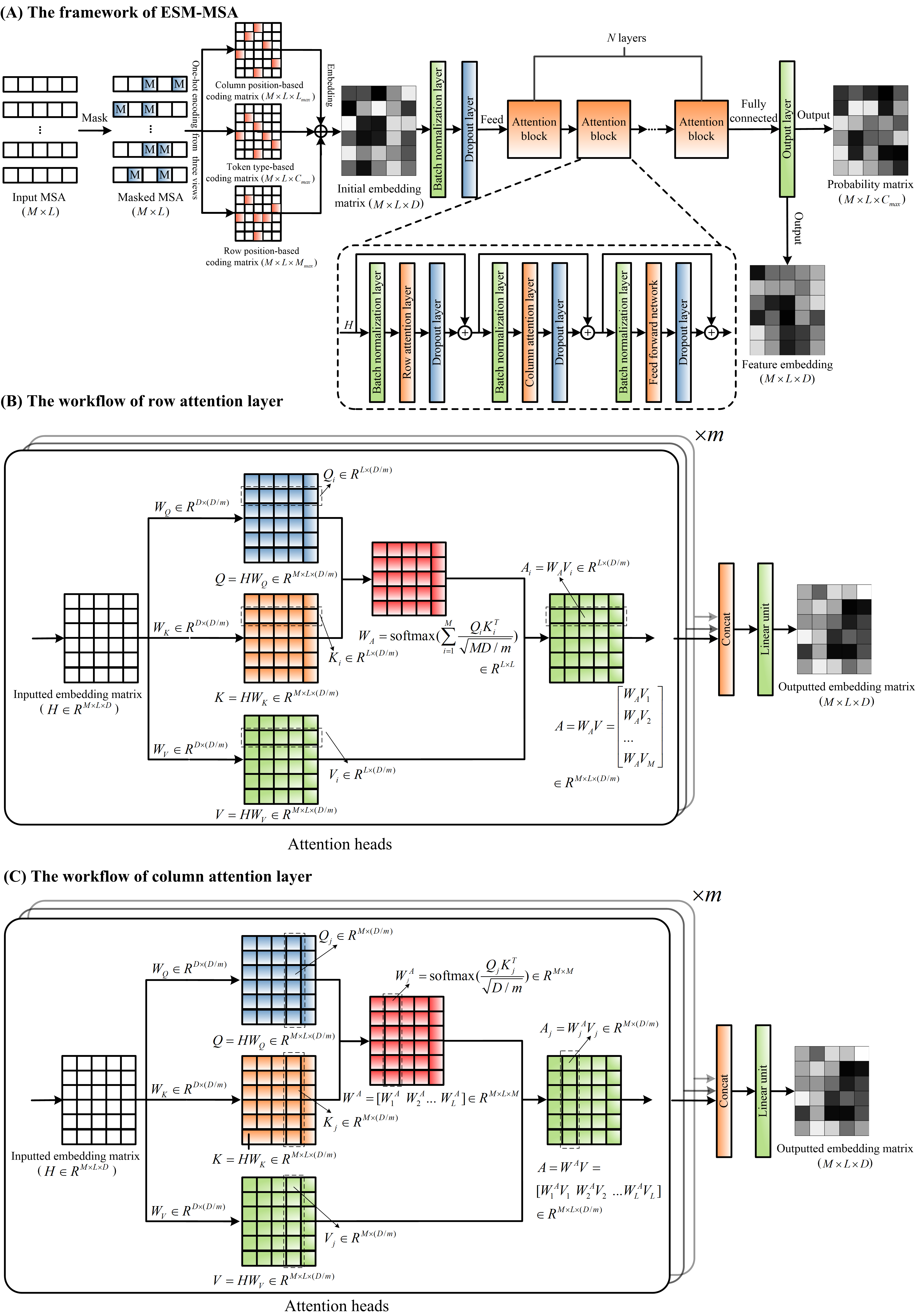


Figure S3. The workflow of the ESM-MSA transformer.

**
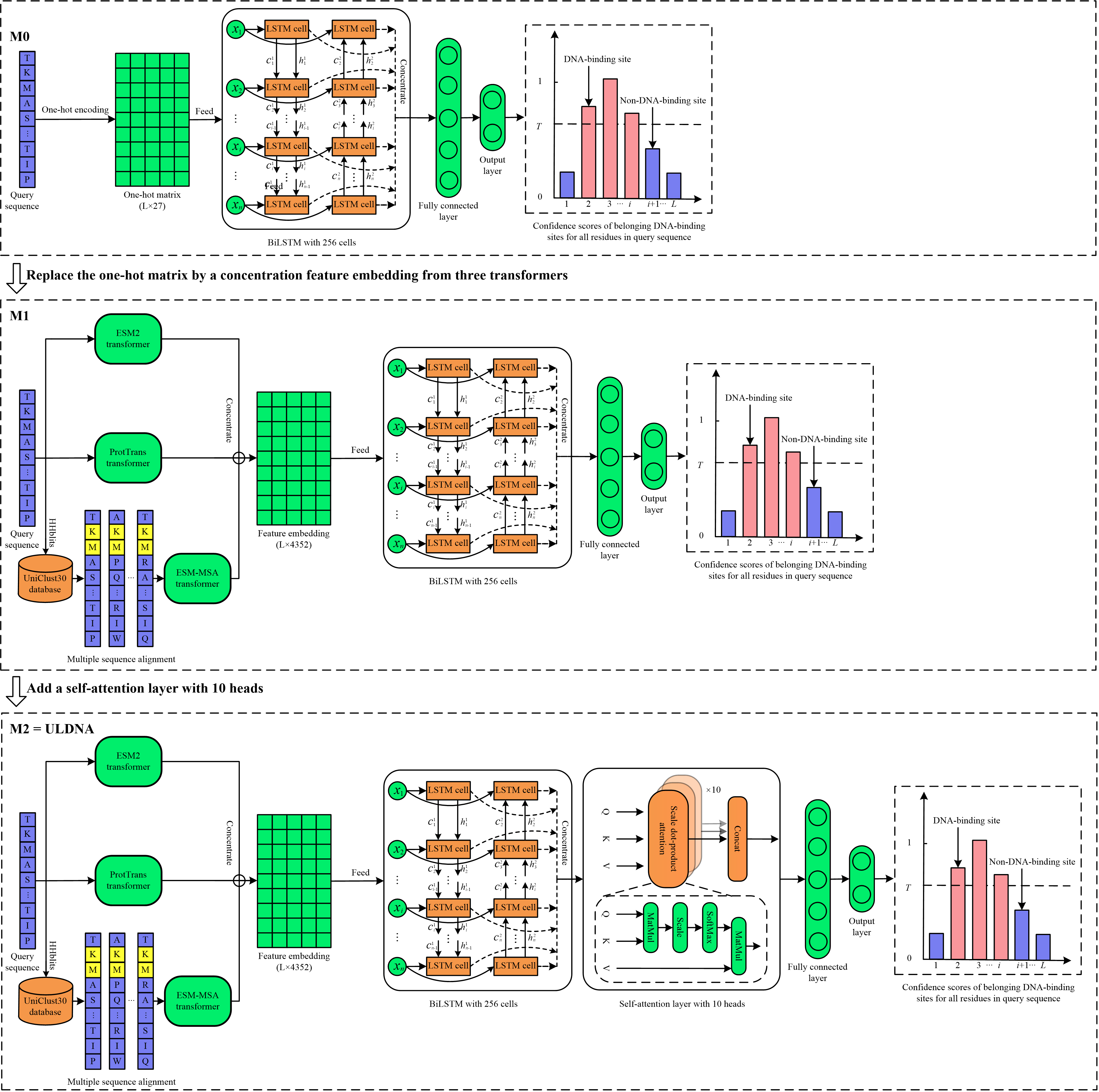
**

Figure S4. The architectures of three ablation models

**Supporting Tables**

Table S1. The performance of 5 DNA-binding site predictors

on the PDNA-543 dataset over ten-fold cross-validation.

| Method | Sen | Spe | Acc | MCC | AUROC |
| --- | --- | --- | --- | --- | --- |
| TargetDNA ($Sen\approx Spe$) ^a^ | 0.770 | 0.771 | 0.770 | 0.304 | 0.845 |
| DNAPred ($Sen\approx Spe$) ^b^ | 0.771 | 0.785 | 0.784 | 0.318 | 0.861 |
| PredDBR ($Sen\approx Spe$) ^c^ | 0.776 | 0.774 | 0.774 | 0.338 | - |
| ULDNA ($Sen\approx Spe$) | **0.864** | **0.861** | **0.861** | **0.462** | **0.933** |
| TargetDNA ($Spe\approx0.95$) ^a^ | 0.406 | 0.950 | 0.914 | 0.339 | 0.845 |
| DNAPred ($Spe\approx0.95$) ^b^ | 0.449 | 0.950 | 0.917 | 0.373 | 0.861 |
| PredDBR ($Spe\approx0.95$) ^c^ | 0.465 | 0.950 | 0.911 | 0.409 | - |
| Guan’s method ($Spe\approx0.95$) ^d^ | 0.452 | **0.954** | 0.928 | 0.352 | - |
| ULDNA ($Spe\approx0.95$) | **0.668** | 0.950 | **0.931** | **0.534** | **0.933** |

^a, b, c, d^ Results excerpted from TargetDNA [6], DNAPred [12], PredDBR [13], and Guan et al [14], respectively. “$Sen\approx Spe$” and “$Spe\approx0.95$” mean that the thresholds make $Sen\approx Spe$ and “$Spe\approx0.95$”, respectively, on the PDNA-543 dataset over ten-fold cross-validation. ‘-’ means the value is not available. Bold fonts highlight the best performer in each evaluation index.

Table S2. The performance of 5 DNA-binding site predictors

on the PDNA-335 dataset over ten-fold cross-validation.

| Method | Sen | Spe | Acc | MCC | AUROC |
| --- | --- | --- | --- | --- | --- |
| EC-RUS ^a^ | 0.487 | 0.951 | **0.926** | 0.378 | 0.852 |
| TargetS ^b^ | 0.417 | 0.945 | 0.899 | 0.362 | 0.824 |
| DNAPred ^c^ | 0.543 | 0.917 | 0.886 | 0.390 | 0.856 |
| PredDBR ^d^ | 0.426 | **0.953** | 0.910 | 0.390 | - |
| ULDNA | **0.676** | 0.948 | 0.925 | **0.565** | **0.940** |

^a, b, c, d^ Results excerpted from EC-RUS [15], TargetS [16], DNAPred [12], and PredDBR [13], respectively. ‘-’ means the value is not available. Bold fonts highlight the best performer in each evaluation index.

Table S3. The performance of 11 DNA-binding site predictors

on the PDNA-316 dataset over ten-fold cross-validation.

| Method | Sen | Spe | Acc | MCC |
| --- | --- | --- | --- | --- |
| DBS-PRED ^a^ | 0.530 | 0.760 | 0.750 | 0.170 |
| BindN ^a^ | 0.540 | 0.800 | 0.780 | 0.210 |
| DNABindR ^a^ | 0.660 | 0.740 | 0.730 | 0.230 |
| DISIS ^a^ | 0.190 | 0.980 | 0.920 | 0.250 |
| DP-Bind ^a^ | 0.690 | 0.790 | 0.780 | 0.290 |
| BindN-rf ^a^ | 0.670 | 0.830 | 0.820 | 0.320 |
| MetaDBSite ^a^ | 0.770 | 0.770 | 0.770 | 0.320 |
| TargetDNA ($Sen\approx Spe$) ^a^ | 0.780 | 0.780 | 0.780 | 0.339 |
| TargetDNA ($Spe\approx0.95$) ^a^ | 0.430 | 0.950 | 0.910 | 0.375 |
| DNAPred ($Sen\approx Spe$) ^b^ | 0.800 | 0.799 | 0.799 | 0.370 |
| DNAPred ($Spe\approx0.95$) ^b^ | 0.521 | 0.951 | 0.918 | 0.452 |
| PredDBR ($Sen\approx Spe$) ^c^ | 0.815 | 0.807 | 0.808 | 0.398 |
| PredDBR ($Spe\approx0.95$) ^c^ | 0.561 | 0.953 | 0.921 | 0.497 |
| PredDBR (threshold = 0.5) ^c^ | 0.531 | 0.958 | 0.923 | 0.489 |
| ULDNA ($Sen\approx Spe$) | **0.871** | 0.867 | 0.867 | 0.502 |
| ULDNA ($Spe\approx0.95$) | 0.676 | 0.950 | 0.929 | 0.561 |
| ULDNA (threshold = 0.5) | 0.449 | **0.983** | **0.942** | **0.526** |

^a, b, c^ Results excerpted from TargetDNA [6], DNAPred [12], and PredDBR [13], respectively. “$Sen\approx Spe$” and “$Spe\approx0.95$” mean that the thresholds make $Sen\approx Spe$ and “$Spe\approx0.95$”, respectively, on the PDNA-316 dataset over ten-fold cross-validation. Bold fonts highlight the best performer in each evaluation index.

Table S4. The *p*-values of MCC values between EPE and the other six feature embeddings on five benchmark datasets, where the base model is the designed LSTM-attention network.

| Dataset | Feature embeddings from different protein language models | | | | | |
| --- | --- | --- | --- | --- | --- | --- |
|  | (EPE, ESM2) | (EPE, ProtTrans) | (EPE, ESM-MSA) | (EPE, PE) | (EPE, EE) | (EPE, EP) |
| PDNA-543 | 1.96×10^-06^ | 2.96×10^-12^ | 3.43×10^-12^ | 1.01×10^-08^ | 4.25×10^-04^ | 9.55×10^-04^ |
| PDNA-335 | 3.89×10^-10^ | 7.74×10^-16^ | 1.40×10^-11^ | 6.08×10^-08^ | 1.25×10^-03^ | 6.64×10^-06^ |
| PDNA-316 | 1.37×10^-09^ | 1.13×10^-10^ | 1.15×10^-14^ | 1.57×10^-07^ | 4.34×10^-06^ | 9.42×10^-03^ |
| PDNA-41 | 8.22×10^-08^ | 9.25×10^-07^ | 5.21×10^-09^ | 2.75×10^-04^ | 1.97×10^-03^ | 5.99×10^-02^ |
| PDNA-52 | 5.82×10^-05^ | 6.39×10^-04^ | 8.78×10^-09^ | 1.12×10^-03^ | 1.91×10^-02^ | 3.14×10^-02^ |

ESM2: the feature embedding from the ESM2 transformer; ProtTrans: the feature embedding from the ProtTrans transformer; ESM-MSA: the feature embedding from the ESM-MSA transformer; PE: the concatenation of two feature embeddings from the ProtTrans and ESM-MSA transformers; EE: the concatenation of two feature embeddings from the ESM2 and ESM-MSA transformers; EP: the concatenation of two feature embeddings from the ESM2 and ProtTrans transformers; EPE: the concatenation of three feature embeddings from the ESM2, ProtTrans, and ESM-MSA transformers.

Table S5. The *p*-values of AUROC values between EPE and the other six feature embeddings on five benchmark datasets, where the base model is the designed LSTM-attention network.

| Dataset | Feature embeddings from different protein language models | | | | | |
| --- | --- | --- | --- | --- | --- | --- |
|  | (EPE, ESM2) | (EPE, ProtTrans) | (EPE, ESM-MSA) | (EPE, PE) | (EPE, EE) | (EPE, EP) |
| PDNA-543 | 1.14×10^-08^ | 5.02×10^-10^ | 5.84×10^-08^ | 1.06×10^-05^ | 3.40×10^-02^ | 9.89×10^-05^ |
| PDNA-335 | 1.76×10^-10^ | 8.12×10^-13^ | 1.45×10^-12^ | 1.38×10^-11^ | 2.67×10^-05^ | 4.52×10^-07^ |
| PDNA-316 | 1.77×10^-08^ | 8.99×10^-11^ | 1.77×10^-12^ | 1.31×10^-08^ | 6.42×10^-05^ | 1.85×10^-07^ |
| PDNA-41 | 2.28×10^-07^ | 1.37×10^-03^ | 5.84×10^-05^ | 7.17×10^-02^ | 3.48×10^-01^ | 2.37×10^-02^ |
| PDNA-52 | 3.77×10^-03^ | 2.40×10^-04^ | 4.47×10^-06^ | 9.24×10^-04^ | 6.44×10^-03^ | 2.07×10^-01^ |

ESM2: the feature embedding from the ESM2 transformer; ProtTrans: the feature embedding from the ProtTrans transformer; ESM-MSA: the feature embedding from the ESM-MSA transformer; PE: the concatenation of two feature embeddings from the ProtTrans and ESM-MSA transformers; EE: the concatenation of two feature embeddings from the ESM2 and ESM-MSA transformers; EP: the concatenation of two feature embeddings from the ESM2 and ProtTrans transformers; EPE: the concatenation of three feature embeddings from the ESM2, ProtTrans, and ESM-MSA transformers.

Table S6. The predicted and native DNA-binding sites of two representative proteins

for five DNA-binding prediction methods.

| Protein | Method | Predicted DNA-binding sites |
| --- | --- | --- |
| 2MXF_A | LA-ESM2 | **2R 5K** **7Y** 10P 11H **18T 19K** **20G 21G 22N 23H 24K 27K 30K** |
|  | LA-ProtTrans | **1A** **2R** **3K** 4V **5K** 16I 17E **18T 19K 20G 21G 22N** **23H 24K** 25T **27K** |
|  | LA-ESM-MSA | **2R 5K** **7Y 18T 19K 20G 21G 22N 23H 24K 27K 30K** 41W |
|  | ULDNA | **2R 3K 5K 7Y 18T 19K 20G 21G 22N 23H 24K 27K 30K** |
|  | PredDBR | **2R 5K 7Y** 8K 9N **18T 19K 20G 21G 23H** |
|  | Native DNA-binding sites | 1A 2R 3K 5K 7Y 18T 19K 20G 21G 22N 23H 24K 26L 27K 30K 39E |
| 3ZQL_A | LA-ESM2 | **12R** 13R 14S 15A 16R 17S 18H **19R** 20T **43S 44M 45R 54G 55T 56M 57S 59Y 60Y 61Y** 180R 183 M |
|  | LA-ProtTrans | 13R 14S 15A 16R 17S 18H **19R** 20T 21L **43S** **44M 45R 55T 56M 57S 59Y 60Y 61Y 65K** |
|  | LA-ESM-MSA | **12R** 13R 14S 15A 16R 17S 18H **19R** 20T 23R **43S 44M 45R** 46R 53A **54G 55T 56M** **57S 59Y 60Y 61Y 64T 65K** |
|  | ULDNA | **12R** 13R 14S 15A 16R 17S 18H **19R** 20T **43S 44M 45R** 53A **54G 55T 56M 57S 59Y** **60Y 61Y 64T 65K** |
|  | PredDBR | 1V 4W 6H 7P **12R** 15A 18H **19R** 22S 23R **43S 44M 45R** 53A **54G 55T 56M 57S 59Y**  **60Y 61Y 64T 65K** 115W 117N 119H 124P 125N 126S 182W 187G 236D |
|  | Native DNA-binding sites | 12R 19R 43S 44M 45R 54G 55T 56M 57S 59Y 60Y 61Y 64T 65K |

Bold font means a DNA-binding site can be correctly predicted by a DNA-binding prediction method.

**Reference**

[1] J. Yang, A. Roy, and Y. Zhang, "BioLiP: a semi-manually curated database for biologically relevant ligand–protein interactions," *Nucleic acids research,* vol. 41, no. D1, pp. D1096-D1103, 2012.

[2] T. Gallo Cassarino, L. Bordoli, and T. Schwede, "Assessment of ligand binding site predictions in CASP10," *Proteins: Structure, Function, and Bioinformatics,* vol. 82, pp. 154-163, 2014.

[3] T. Schmidt, J. Haas, T. G. Cassarino, and T. Schwede, "Assessment of ligand‐binding residue predictions in CASP9," *Proteins: Structure, Function, and Bioinformatics,* vol. 79, no. S10, pp. 126-136, 2011.

[4] C. Zhang, X. Zhang, P. L. Freddolino, and Y. Zhang, "BioLiP2: an updated structure database for biologically relevant ligand–protein interactions," *Nucleic Acids Research,* p. gkad630, 2023.

[5] S. Ahmad, M. M. Gromiha, and A. Sarai, "Analysis and prediction of DNA-binding proteins and their binding residues based on composition, sequence and structural information," *Bioinformatics,* vol. 20, no. 4, pp. 477-486, 2004.

[6] J. Hu, Y. Li, M. Zhang, X. Yang, H.-B. Shen, and D.-J. Yu, "Predicting protein-DNA binding residues by weightedly combining sequence-based features and boosting multiple SVMs," *IEEE/ACM Transactions on Computational Biology and Bioinformatics,* vol. 14, no. 6, pp. 1389-1398, 2016.

[7] J. Devlin, M.-W. Chang, K. Lee, and K. Toutanova, "Bert: Pre-training of deep bidirectional transformers for language understanding," *arXiv preprint arXiv:1810.04805,* 2018.

[8] J. Buckman, A. Roy, C. Raffel, and I. Goodfellow, "Thermometer encoding: One hot way to resist adversarial examples," in *International Conference on Learning Representations*, 2018.

[9] D. P. Kingma and J. Ba, "Adam: A method for stochastic optimization," *arXiv preprint arXiv:1412.6980,* 2014.

[10] J. C. De Winter, "Using the Student's t-test with extremely small sample sizes," *Practical Assessment, Research, and Evaluation,* vol. 18, no. 1, p. 10, 2019.

[11] W. Li and A. Godzik, "CD-HIT: a fast program for clustering and comparing large sets of protein or nucleotide sequences," *Bioinformatics,* vol. 22, no. 13, pp. 1658-1659, 2006.

[12] Y.-H. Zhu, J. Hu, X.-N. Song, and D.-J. Yu, "DNAPred: accurate identification of DNA-binding sites from protein sequence by ensembled hyperplane-distance-based support vector machines," *Journal of Chemical Information and Modeling,* vol. 59, no. 6, pp. 3057-3071, 2019.

[13] J. Hu, Y.-S. Bai, L.-L. Zheng, N.-X. Jia, D.-J. Yu, and G.-J. Zhang, "Protein-DNA binding residue prediction via bagging strategy and sequence-based cube-format feature," *IEEE/ACM Transactions on Computational Biology and Bioinformatics,* vol. 19, no. 6, pp. 3635-3645, 2021.

[14] S. Guan, Q. Zou, H. Wu, and Y. Ding, "Protein-DNA binding residues prediction using a deep learning model with hierarchical feature extraction," *IEEE/ACM Transactions on Computational Biology and Bioinformatics,* p. DOI: 10.1109/TCBB.2022.3190933, 2022.

[15] Y. Ding, J. Tang, and F. Guo, "Identification of protein-ligand binding sites by sequence information and ensemble classifier," *Journal of Chemical Information and Modeling,* vol. 57, no. 12, pp. 3149-3161, 2017.

[16] D.-J. Yu, J. Hu, J. Yang, H.-B. Shen, J. Tang, and J.-Y. Yang, "Designing template-free predictor for targeting protein-ligand binding sites with classifier ensemble and spatial clustering," *IEEE/ACM Transactions on Computational Biology and Bioinformatics,* vol. 10, no. 4, pp. 994-1008, 2013.
